# Fractal gradient divergence-tuned deep belief network for osteoporosis detection using X-ray images

**DOI:** 10.3389/frai.2026.1870392

**Published:** 2026-07-01

**Authors:** M. Raja, Avulapalli Jayaram Reddy

**Affiliations:** School of Computer Science Engineering and Information Systems, Vellore Institute of Technology, Vellore, Tamil Nadu, India

**Keywords:** deep belief network (DBN), gradient divergence crow search tuning algorithm, Grubbs normalized linear box blur method, osteoporosis disease prediction, rotation transformed image SMOTE based augmentation

## Abstract

Osteoporosis is a major illness that reduces bone strength, increasing the likelihood of fractures. Various imaging modalities are employed in the prediction and diagnosis of osteoporosis, including X-ray and Computed Tomography (CT). To assess the risk of fractures and bone disease, various machine learning (ML) techniques have been utilized. However, accurate diagnosis based on X-ray images remains a major concern for osteoporosis prediction. To improve the accuracy of osteoporosis disease prediction and reduce time, a novel Transformed Sampling Fractal Gradient Divergence Tuned Deep Belief Network (TSFGDTDBN) is proposed. It includes image acquisition, Augmentation, preprocessing, feature extraction, classification, and fine-tuning. Initially, numerous X-ray knee images were collected from the dataset during the acquisition phase. Rotation-transformed Image-SMOTE-based augmentation was employed for creating synthetic image samples of the minority class. The Gradient Divergence Tuned Deep Belief Network (DBN) involves two primary steps: layer-by-layer training and fine-tuning. In layer-by-layer training, preprocessing is performed with the Grubbs-normalized linear box blur method. Edge, shape, and texture features are extracted. Finally, the disease is classified as normal, Osteopenia, and Osteoporosis. During fine-tuning, hyperparameters are optimally adjusted using the Gradient Divergence Crow Search Tuning algorithm to improve classification accuracy. It enhances the deep neural network's performance and overall learning efficiency in osteoporosis disease prediction. Experimental evaluation is conducted using X-ray images with various factors. TSFGDTDBN improves accuracy with minimal time compared to conventional deep learning methods.

## 1 Introduction

Osteoporosis is a medical condition characterized by reduced bone density in older individuals and postmenopausal women. Timely screening and evaluation of osteoporosis risk are essential to prevent fragility fractures. In the diagnosis of osteoporosis, various imaging techniques are utilized. Among these, X-ray imaging has provided valuable insights into bone structure and density. Recently, several methods using ML and DL have been developed to support the diagnosis of osteoporosis through automated image analysis and pattern recognition. DenseNet201 with transfer learning was introduced in Harris et al. (2025) to accurately distinguish osteoporosis from healthy bones in X-ray images. The designed model enhances diagnostic accuracy while minimizing the time required to optimize performance in osteoporosis classification. A VGG-19 deep learning model was developed in Sarhan et al. (2024) to improve the accuracy and efficiency of osteoporosis detection. However, the model failed to perform hyperparameter optimization to improve osteoporosis prediction without compromising computational complexity. A vision transformer was employed in Huang et al. (2026) to categorize hip X-ray images, thereby enhancing accuracy. However, the time was not minimized. Through optimization techniques, deepCNNs were utilized (Diab et al., 2025). However, achieving consistent improvements across diverse datasets and clinical settings remains a significant challenge. In Zhang et al. (2023), a joint learning framework was designed. However, efficient deep learning models were not implemented to further enhance model accuracy on osteoporosis diagnosis by analyzing and capturing complex features. In Naguib et al. (2024b), transfer learning was developed. However, the model failed to apply more advanced optimization techniques. A weighted ensemble approach was designed in Rasool et al. (2024) to differentiate the normal and osteoporotic knee conditions. However, the model's accuracy did not improve. A non-invasive computer-aided diagnosis system was designed in El-Sisi et al. (2025) that uses a deep learning model to predict Osteopenia and Osteoporosis from knee X-ray images. However, the optimization model was not applied to improve the computational efficiency.

In Mewada et al. (2025), bone fractures from X-Ray images were detected via Quantum CNN. However, class imbalance was not addressed to improve accuracy. A fully automated convolutional neural network (CNN) model was developed in Chen et al. (2024) to differentiate osteoporosis diseases. However, it did not achieve significant improvements in the early diagnosis of osteoporosis within a minimal time. A novel method was introduced in Higuchi et al. (2025) to efficiently detect osteoporosis from X-ray images. However, its precision did not show significant improvement in osteoporosis detection. A machine learning (ML)-based predictive model was introduced in Siddharth et al. (2025) for predicting osteoporosis using demographic and clinical data. However, the model failed to perform feature-importance analysis for prediction. Machine learning (ML) and explainable artificial intelligence (XAI) models were developed by Khanna et al. (2023) for osteoporosis prediction. However, the time complexity was not reduced effectively. In Zhao et al. (2025), novel deep-learning (DL) techniques were introduced. However, it did not effectively reduce the misidentification of osteoarthritis. In Li et al. (2025), the prevalence of osteoporosis and osteopenia was analyzed. However, the model failed to learn deep features for osteoporosis prediction. In Rehman et al. (2023), an advanced deep learning basis of CNN was developed. Nevertheless, the augmentation scheme was not employed.

### 1.1 Novelty and contribution

The novel contributions of the TSFGDTDBN model are described as follows,

- TSFGDTDBN model is developed for obtaining the osteoporosis disease prediction by incorporating data collection, data augmentation, preprocessing, feature extraction, and classification into the Deep Belief Network.
- Rotation transformed SMOTE is employed in the image dataset to perform data augmentation for creating the data samples, providing high-quality image samples.
- A Deep Belief Network (DBN) is utilized in the proposed method, with several layers, to analyze features more deeply and achieve higher accuracy in less time.
- Grubbs normalized linear box blur method is applied for image preprocessing to eradicate the noisy images. Geometric features such as edge, fractal dimension, and texture are extracted by using the Marr–Hildreth edge detector. Furthermore, the Minkowski-Bouligand fractal geometric method is used to extract the fractal dimension of healthy bone.
- Rand similarity index function is employed in the TSFGDTDBN model to perform Osteoporosis classification for investigating the similarity between the extracted features and testing feature value for normal, Osteopenia, and osteoporosis with higher precision and lesser detection time.
- Gradient Divergence Crow Search Tuning algorithm is employed at TSFGDTDBN for executing the fine-tuning to determine the optimal weight via fitness with minimum classification error. Multiclass prediction results are obtained through softmax activation.
- TSFGDTDBN utilized to execute experimental evaluation with several factors compared with other deep learning methods.

### 1.2 Organization

Manuscript prearranged by: Literature survey discussed in Section 2. TSFGDTDBN is presented in Section 3, accompanied by a comprehensive diagram. Section 4 outlines the experimental setup and describes the dataset, while Section 5 presents a comparative analysis of various factors. Finally, the conclusion is portrayed in Section 6.

## 2 Related works

Osteoarthritis was recognized in Rani et al. (2024) through a 12-layer CNN. In Wang et al. (2025), attention-enhanced autoencoders were developed. Nevertheless, it failed to achieve both scalability and accuracy for clinical and medical applications. An ML model was developed in Je et al. (2025) using data from a large-scale population cohort study. However, this model did not account for the larger dataset volume in osteoporosis classification. A vision transformer model was developed in Sarmadi et al. (2024) for detecting osteoporosis. In Amjad et al. (2025), an end-to-end deep learning architecture was introduced for the precise detection of knee osteoarthritis (KOA) disease. Feature extraction was performed using an autoencoder. Images were classified by using Extreme Learning Machines (ELM). However, it failed to consider recall. A spectral domain analysis was introduced in Dhanyavathi and Veena (2023) by using a two-dimensional discrete wavelet transform. However, early detection of osteoporosis was a major issue. The DL model was developed in Feng et al. (2023) to predict osteoporosis. However, it failed to develop a custom model to predict osteoporosis from X-ray images. A deep learning model was designed in Jiang et al. (2024) to design a lossless compression network for high-quality osteoporosis diagnosis using X-ray images. However, the time complexity was not minimized. Two cutting-edge convolutional neural network architectures were proposed in Shawon et al. (2024) for the automatic recognition of osteoporosis. However, it is a field aimed at improving the early detection of osteoporosis. Residual Neural Networks were designed in Mohammed et al. (2023) for identifying the presence or absence of KOA. However, the prediction time was higher. Sensitivity and specificity were enhanced (Dhanagopal et al., 2024) using a transfer-learning CNN. Furthermore, it failed to improve image accuracy and did not effectively eliminate the noise from the image. In Kim (2024), Multivariate logistic regression and extreme gradient boosting methods were developed. In SK and Areeckal (2025), ML models were constructed to categorize healthy images from osteoporotic images. However, pertinent features were not extracted. A multi-view CT network model was developed in Hwang et al. (2023) using X-ray images. However, the model was inefficient for 3D medical imaging. In Niu et al. (2023), a DL system was developed. However, this model did not apply to X-ray image datasets. Hybrid Deep Learning Models were investigated in Alsahafi et al. (2026) for lesion categorization. However, the accuracy was not enhanced. A deep learning method was employed in Hosny et al. (2024) to identify the seven types of skin lesions. Yet another Residual Deep Convolutional Neural Network was analyzed in Alsahafi et al. (2023) to extract multilevel features of skin lesions. But the time was not considered. An XAI framework was analyzed in Kassem et al. (2023) for detecting pelvic fractures. However, the recall was not increased. A computer-aided diagnosis system was used in Naguib et al. (2023) to estimate the spatial support of an exact class. However, the error rate was not measured. A deep learning model was analyzed in Naguib et al. (2024a) to detect uni- or bicompartmental knee OA using a redefined residual CNN. However, it failed to select the relevant features in a shorter time. Transfer learning using two pre-trained models was proposed in Naguib et al. (2024b) for identifying knee osteoporosis and osteopenia in X-ray images. However, the time was higher.

## 3 Proposal methodology

**Figure 1:**
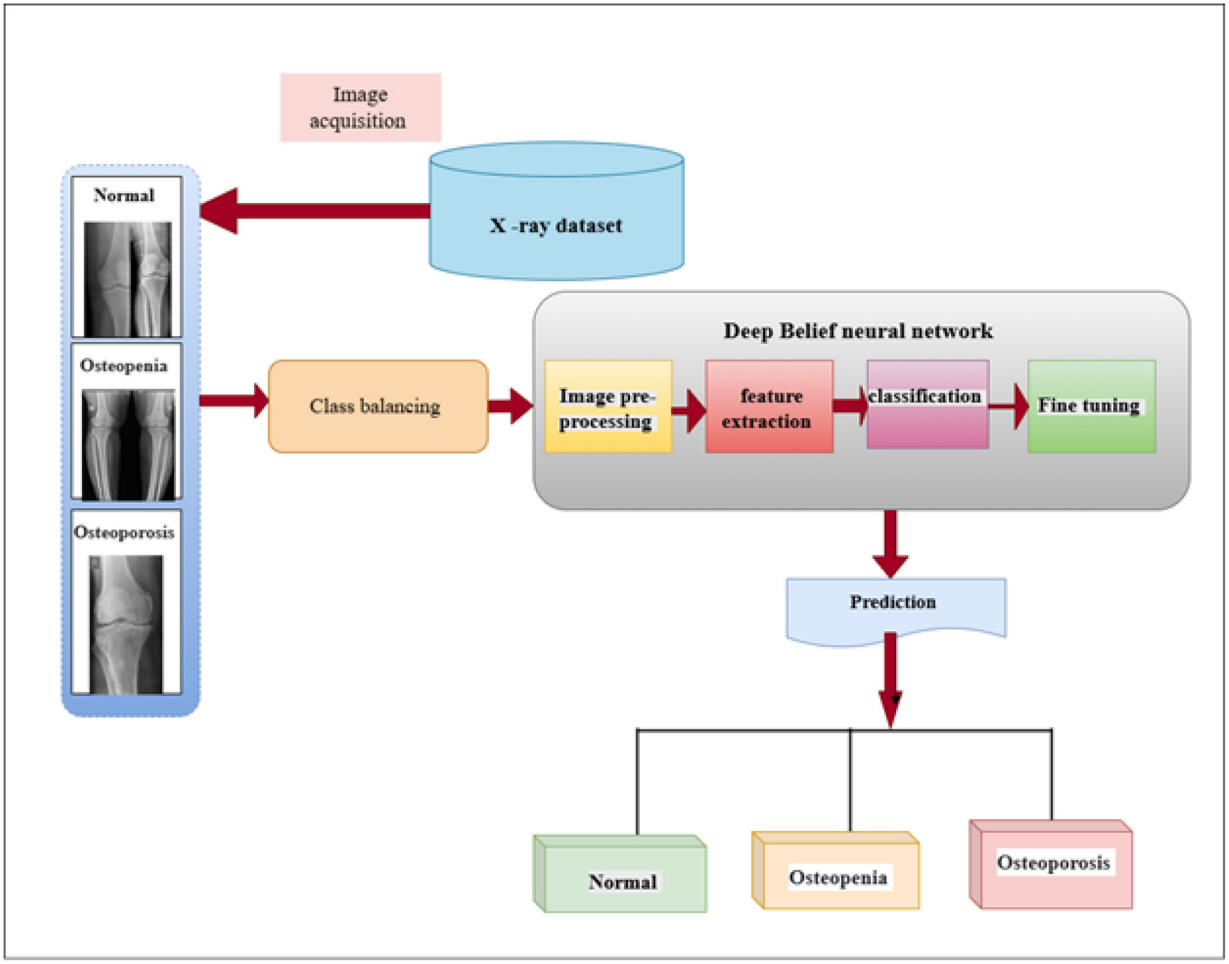
Structural overview of the TSFGDTDBN model for osteoporosis prediction. Diagram created by the authors using X-ray samples from the MultiClass Knee Osteoporosis X-Ray Dataset (https://www.kaggle.com/datasets/mohamedgobara/multi-class-knee-osteoporosis-x-ray-dataset).

Osteoporosis is a generalized bone condition characterized by a significant reduction in bone density. It is characterized by the most important public health problems and the most common bone diseases. Therefore, accurate and timely prediction is essential for effective treatment and management. In this study, a novel TSFGDTDBN model is developed for the accurate and timely prediction of osteoporosis disease using X-ray knee images. The proposed TSFGDTDBN is used for Osteoporosis prediction and provides multi-class results, including Normal, Osteopenia, and Osteoporosis, with minimal time and higher accuracy. Figure 1 shows TSFGDTDBN.

Structure of TSFGDTDBN is illustrated in Figure 1 for accurate osteoporosis disease prediction. The proposed method comprises six fundamental steps: image acquisition, class balancing, pre-processing, feature extraction, classification, and fine-tuning. First, X-ray images are acquired from the Knee Osteoporosis X-Ray Dataset. Data augmentation is employed to address the class-balancing issue. Image preprocessing plays an important role in reducing noise and increasing image quality by using the Grubbs-normalized linear box blur method. The feature extraction process is used in the Marr–Hildreth edge detector and the Minkowski Bouligand fractal geometric method to extract the pertinent features from the database. Osteoporosis classification is performed using the Rand similarity index to classify images as normal, Osteopenia, or osteoporosis. Finally, fine-tuning is performed using the Gradient Divergence Crow Search Tuning algorithm to identify the optimal weight. Multiclass prediction results are achieved during softmax activation. These fundamental steps are integrated into the deep learning network architecture to enhance the accuracy of osteoporosis prediction and reduce processing time. The detailed process of the proposed TSFGDTDBN model is explained briefly in the following subsections.

### 3.1 Image acquisition

It involves collecting multiple image samples to aid in the timely detection and prediction of osteoporosis. In this phase, the Multi-Class Knee Osteoporosis X-Ray Dataset is taken from https://www.kaggle.com/datasets/mohamedgobara/multi-class-knee-osteoporosis-x-ray-dataset. This dataset is used in the research field, such as Knee Osteoporosis Diagnosis Based on DL. The dataset comprises 1947 X-ray images. The image format is JPG or PNG format, and the size is 1.3GB. The dataset's native resolutions vary by source. For deep learning compatibility, they are frequently preprocessed and resized into standardized formats (224 × 224 pixels). X-ray radiographs are monochrome by nature and use both Grayscale (single-channel) and RGB (three-channel) formats. It comprises medical imaging data, specifically X-rays, and related patient records that are essential for diagnosing knee osteoporosis. Labels are frequently derived from proxy clinical information, such as Quantitative Ultrasound (QUS) T-scores and physician annotations. Osteoporosis predominantly targets older populations and postmenopausal women. ML studies that leverage this dataset are largely tailored to these high-risk demographics. Clinical elements such as Age, Gender, Menopause age, Comorbidities (diabetes, thyroid issues), Fracture history, and lifestyle habits (diet, physical activity) are considered in this dataset. The dataset is partitioned into three major categories: normal, Osteopenia, and Osteoporosis, as shown in Table 1.

**Table 1:** Dataset description before augmentation.

Three classes	Description	Number of images before augmentation
Normal	Images of knees with no signs of osteoporosis	780
Osteopenia	Images showing early stages of bone density loss	374
Osteoporosis	Images indicating advanced bone density degradation	793
Total images		1,947

Yet another dataset, the Knee X-ray Osteoporosis Database, was used to conduct the experiments. The dataset is obtained from https://www.kaggle.com/datasets/orvile/knee-x-ray-osteoporosis-database. The database aims to support research in developing accessible, cost-effective systems for early detection of osteoporosis using knee X-ray images and associated clinical data. Osteoporosis is a general bone disease that affects millions worldwide. The dataset comprises images of normal, Osteopenia, and osteoporosis. The dataset consists of 241 files and 28 columns. This dataset supports data-driven research by providing a well-structured collection of knee X-ray images and clinical indicators, enabling the development of AI-powered diagnostic tools. High-quality X-ray images of knee joints were considered from several participants. Clinical factors such as Age, Gender, Menopause age, Comorbidities (diabetes, thyroid issues), Fracture history, and lifestyle habits (diet, physical activity) are included. Using two datasets, cross-validation is used to assess the model's generalization capability and avoid overfitting on unseen data. Using cross-validation, the dataset is divided into training, testing, and validation sets. Most X-ray images (70%) were used for training, a smaller number (20%) for testing, and the remaining (10%) for validation. In our work, 10-fold cross-validation is used to evaluate the results.

### 3.2 Rotation transformed Image-SMOTE based augmentation

In the proposed TSFGDTDBN, the class imbalance issue is addressed after image acquisition. It refers to a condition in classification tasks where class allocation in the dataset is extremely uneven, with minority classes having significantly fewer image samples than the majority class. As a result, models may achieve high overall accuracy while performing poorly on the minority class, which is more important to solve the class imbalance in medical diagnoses.

The proposed TSFGDTDBN model utilizes the Rotation-transformed SMOTE (Synthetic Minority Over-sampling Technique), a widely used method for handling class imbalance in classification tasks by creating synthetic samples of the minority class rather than simply duplicating existing ones. Synthetic images are created to perform oversampling tasks.

By randomly selecting minority-class images from the total “*n*” images, the minority class has been oversampled.


$$\begin{array}{l} I_{MC}=I_{1},I_{2},I_{3},\ldots ,I_{k} \end{array}$$ (1)

In the above Equation 1, where *I*_*MC*_ denotes randomly considering the *k* minority class images from the original dataset *DS*. Then, apply a rotation transformation to the selected minority-class images in two-dimensional Cartesian space to obtain the synthetic images. The main aim of rotation is to introduce different variations while preserving its structural integrity. The rotation process is achieved with respect to an angle θ in the two-dimensional space (x,y) as given below in Equation 2,


$$\begin{array}{l} R=\left[\begin{array}{cc} \cos\theta & -\sin\theta \\ \sin\theta & \cos\theta \end{array}\right]\left[\begin{array}{c} x_{\text{old}}\\ y_{\text{old}} \end{array}\right] \end{array}$$ (2)

$$\begin{array}{l} x=x_{\text{old}}\cos\theta -y_{\text{old}}\sin\theta \end{array}$$ (3)

$$\begin{array}{l} y=x_{\text{old}}\sin\theta +y_{\text{old}}\cos\theta \end{array}$$ (4)

Where *R* indicates a rotation matrix, (*x, y*) denotes a newly generated position of the image in two-dimensional space, *x*_*old*_ (Equation 3) and *y*_*old*_ (Equation 4) denote an old position of the image in two-dimensional space, θ indicates a rotation angle. After that, the mean and standard deviation are computed for each rotated image in the following Equations 5 and 6 respectively.


$$\begin{array}{l} \mu_{ij}=\frac{1}{k}\overset{k}{\underset{i=1}{\sum }}I_{i}\left(i,j\right) \end{array}$$ (5)

$$\begin{array}{l} \sigma_{ij}=\sqrt{\frac{1}{k}\overset{k}{\underset{i=1}{\sum }}{\left(I_{i}\left(i,j\right)-\mu_{ij}\right)}^{2}} \end{array}$$ (6)

Where, μ_*ij*_ denotes the mean pixel value at pixel position (*i, j*) across the rotated *k* images, *I*_*i*_(*i, j*) indicates the pixel value at position (*i, j*) in the *k*^*th*^ image, and *k* represents the number of selected minority class images. and σ_*ij*_ indicates the deviation pixel value at pixel position (i,j).

Gaussian noise is added to generate a synthetic minority image *I*_*synthetic*_ for each selected minority class image in following Equation 7.


$$\begin{array}{l} I_{\text{synthetic}}=I_{k}\left(P\right)+\text{GN}\left(0,1\right) \end{array}$$ (7)

Where *I*_*k*_(*P*) denotes a pixel value in the original minority image, Gaussian noise drawn through 0(μ) and 1(σ) value is *GN*(0, 1). Image pixels are normalized to ensure that the image stays within the valid range [0 − 255].


$$\begin{array}{l} P_{\text{synthetic}}\left(N\right)=\frac{P_{\text{synthetic}}-\min_{P}}{\max_{P}-\min_{P}}\times 255 \end{array}$$ (8)

Where, *P*_*synthetic*_(*N*) (Equation 8) denotes a normalized synthetic image pixels scaled up to [0 − 255], *P*_*synthetic*_ denotes pixel value in the synthetic images before normalization, minimum pixel value at image is *min*_*P*_, maximum pixel range at image is *max*_*P*_. This process is performed for all pixels in the input image. Finally, the synthetic images are obtained through the proposed oversampling method.

Algorithm 1 given above illustrates the process of image oversampling using the rotation-transformed Image-SMOTE-based Augmentation method. The augmentation process starts by applying an image dataset as input. A SMOTE selects the minority classes of images from the input dataset. Subsequently, the rotation process is carried out to transform the input minority-class image. After that, Gaussian noise is added to the transformed image. Finally, the normalization process is performed to ensure that all pixels are scaled to the specified range. Through this approach, high-quality image samples are produced. The result is an augmented and balanced image dataset that improves minority-class representation.

Algorithm 1 Rotation transformed Image_SMOTE based augmentation. 
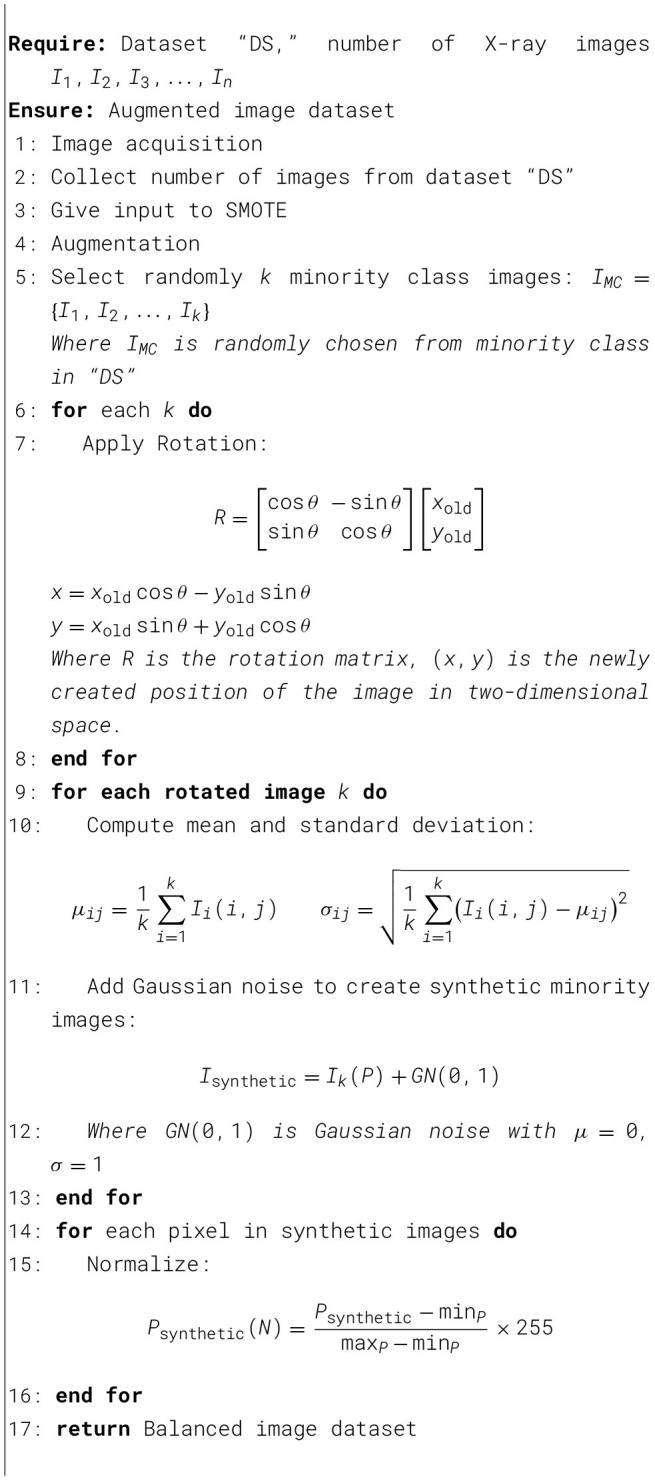

### 3.3 Gradient divergence-tuned deep belief network-based prediction

**Figure 2:**
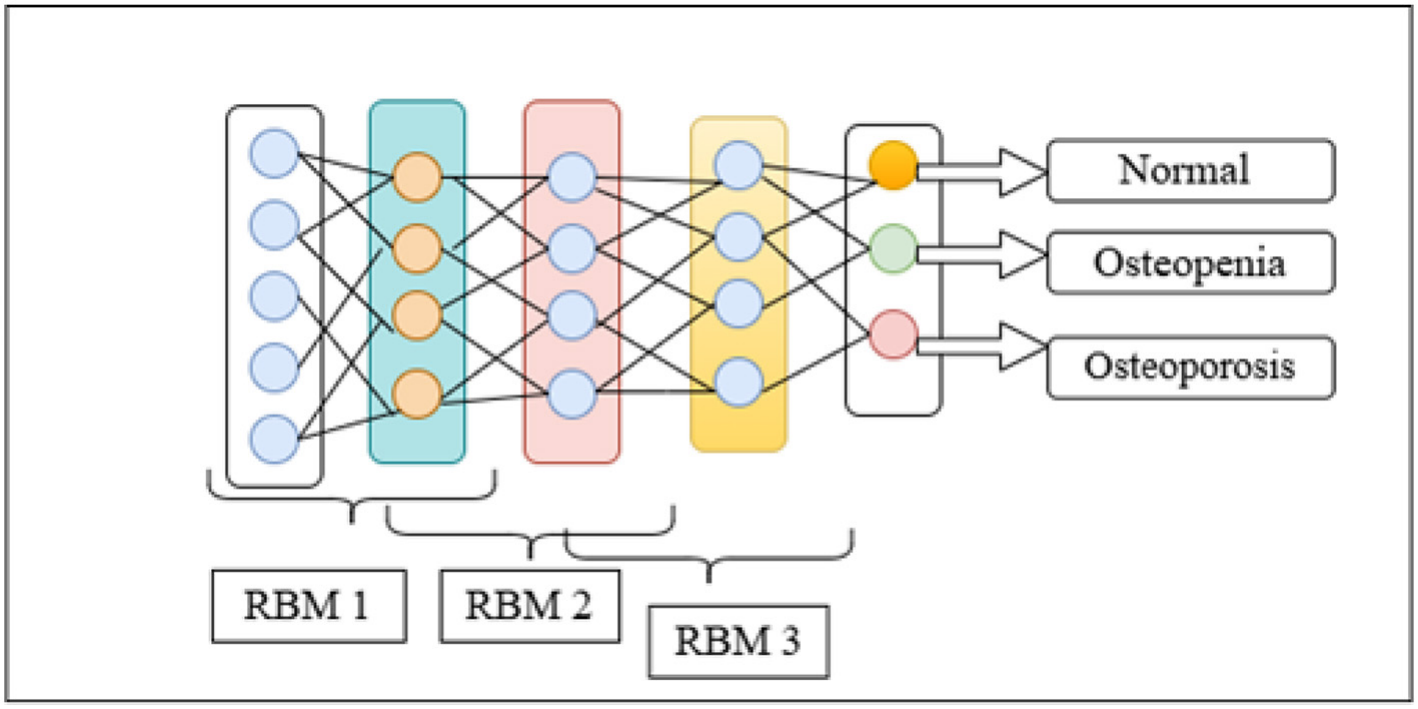
Fundamental construction of DBN.

Deep Belief Network (*DBN*) has an artificial neural network with many layers, including two visible layers, such as an input layer and an output layer, as well as several hidden layers used to process the given input. Training a DBN typically involves two main steps, namely, first, a layer-by-layer pre-training phase, and second, a fine-tuning phase to optimize the entire network. The overall structure of the DBN is shown in Figure 2.

The architecture of DBN is designed in Figure 2 for multiclass classification tasks. The training process involves two main stages. In the layer-by-layer approach, each layer processes weighted input image samples, transforming them before passing them to the next layer. This method forms the backbone of the DBN structure. During the fine-tuning stage, the backpropagation algorithm is used to adjust network hyperparameters, thereby optimizing performance and enhancing osteoporosis prediction accuracy. This optimization not only enhances classification outcomes but also helps minimize errors. The layer-wise pre-training approach employs Restricted Boltzmann Machines (RBMs), which are probabilistic neural networks consisting of a visible layer and a hidden layer that collaboratively process the given image.

In Figure 2, a three-layer RBM (*RestrictedBoltzmannMachine*) architecture is utilized, where each layer is adapted to execute a specific function, such as preprocessing, feature extraction, and classification. The first *RBM* layer is responsible for enhancing the input image quality during preprocessing. The second *RBM* layer processes these enhanced images to extract meaningful features. Finally, the third *RBM* layer uses the extracted features to perform classification, assigning images to their respective predefined categories.

The input X-ray augmented images *I*_1_, *I*_2_, *I*_3_, …, *I*_*h*_ are given to the input visible layer. The neuron in input layer is linked with a weight *w*_1_, *w*_2_, …, *w*_*n*_ and included with bias “*B*.” Therefore, the neuron activation probability *p*_*r*_ of the visible layer is expressed as follows,


$$\begin{array}{l} p_{v}=\varphi \left(\overset{h}{\underset{i=1}{\sum }}I_{i}\cdot w_{i}\right)+B \end{array}$$ (9)

In the above Equation 9, where *p*_*v*_, denotes a neuron activation probability in the visible layer, ϕ denotes a sigmoid activation function, *I*_*i*_ denotes an augmented input X-ray image, *w*_*i*_ denotes weights in the visible layer, and *B* denotes a bias of the visible layer. If the neuron activation probability *p*_*r*_ = 1, then the input image is sent toward the hidden layer. The probability of a neuron in that layer is computed as follows,


$$\begin{array}{l} p_{H}=\varphi \left(\overset{h}{\underset{i=1}{\sum }}I_{i}\cdot w_{ih}\right)+B \end{array}$$ (10)

In the above Equation 10, where *P*_*H*_, indicates a neuron activation probability in the hidden layer, ϕ denotes a sigmoid activation function, *w*_*ih*_ denotes the weights between the visible layer and the hidden layer, and *B* denotes the bias of the hidden layer. The neuron activation probability *p*_*H*_ = 1, and the input is transferred to the next layer.

Image quality is enhanced to execute preprocessing at the first hidden layer. To handle noisy pixels, the Grubbs-normalized linear box blur method is employed. For each input image, the number of pixels *P* is organized in the form of a matrix as follows,


$$\begin{array}{l} Q=\left[\begin{array}{cccc} P_{11} & P_{12} & \cdots & P_{1n}\\ P_{21} & P_{22} & \cdots & P_{2n}\\ \vdots & \vdots & \ddots & \vdots \\ P_{m1} & P_{m2} & \cdots & P_{mn} \end{array}\right] \end{array}$$ (11)

In the above Equation 11, *Q* indicates a matrix where the pixels are organized in rows and columns. Using the above matrix, the Grubbs normalized test is employed to identify noisy pixels in the input image. The Grubbs' normalized test is a statistical method commonly used to identify noisy pixels by assessing whether a given value deviates significantly from the rest of the dataset. The Grubbs normalized test between the pixels is estimated as follows: the statistical test analysis is expressed as follows,


$$\begin{array}{l} NT=\arg\max\left(\frac{P_{j}-\mu_{P}}{\sigma }\right) \end{array}$$ (12)

In the above Equation 12, where *NT* denotes a statistical test outcome, *P*_*j*_ indicates a pixel, μ_*P*_ refers to the mean value of samples, and σ denotes the deviation between the data samples and the mean. From the analysis, the maximum statistical values for the particular pixels are considered noise. Therefore, dataset preprocessing helps improve image quality.

In the Equation 13 below, these noisy pixels are replaced from the input image based on the average of their neighboring pixels.


$$\begin{array}{l} I_{\text{denoised}}=\frac{1}{m}\overset{m}{\underset{j=1}{\sum }}P_{j} \end{array}$$ (13)

Where *I*_*denoised*_ refers to a denoised output, *m* indicates the number of neighboring pixels, and *P*_*j*_ indicates a pixel. As a result, the quality improved, and smoothed X-ray images are observed for accurate disease prediction.

### 3.4 Feature extraction

It is employed in TSFGDTDBN for extracting features such as edges, textures, and shapes from preprocessed images. The proposed technique employs a Marr–Hildreth edge detector in the second hidden layer to detect salient boundaries in X-ray images. This operator enhances the structural details of bone by detecting regions with rapid intensity changes.

This detector utilizes the Laplacian of Gaussian (*LoG*) functions in the spatial domain, where the input preprocessed image is partitioned into uniform pixels with coordinates (*x, y*) at a specific resolution. Therefore, the edge detection is performed as follows,


$$\begin{array}{l} I_{\text{Edge}}=-\frac{1}{\pi \sigma^{4}}\left[1-\frac{x^{2}+y^{2}}{2\sigma^{2}}\right]\cdot \exp\left(-\frac{x^{2}+y^{2}}{2\sigma^{2}}\right) \end{array}$$ (14)

Where *I*_*Edge*_ denotes an edge detection output from the input image,σ indicates a deviation, *x* represents the distance from the origin on the horizontal axis, and *y* refers to the distance from the origin on the perpendicular axis. In Equation 14, the symbol * indicates a convolution operation.

After detecting edges, fractal analysis is performed on the extracted contours to quantify the bone's complexity and structural irregularity. This allows calculation of the fractal dimension, a key feature for distinguishing healthy from osteoporotic bone patterns.

The Minkowski-Bouligand fractal geometric method is applied to evaluate the fractal dimension, which helps characterize the complexity of bone structures in X-ray images and aids accurate osteoporosis detection. The Minkowski Bouligand fractal geometric method involves covering the region of interest (ROI) with a grid of square boxes of varying sizes. For each box size, the number of boxes that intersect with any part of the object (*e*.*g*., bone structure) is counted. This process captures the spatial complexity of the structure across different scales.

Fractal dimension analysis derived from this method is a powerful tool for characterizing structural irregularities. It helps differentiate between normal and osteoporotic tissues by quantifying shape-based complexity. The Fractal dimension analysis is mathematically defined as follows,


$$\begin{array}{l} F\left(S\right)=\underset{c\to 0}{\lim}\left(\frac{\log K\left(c\right)}{\log\left(1/c\right)}\right) \end{array}$$ (15)

In the above Equation 15, where *F*(*S*) indicates a fractal dimension of the structure, *K*(*z*) denotes the number of boxes of size *c* that cover part of the object in the *ROI*, *c* indicates the size of the side length of the box. Healthy bone includes a higher fractal dimension. Osteoporotic Bone has a lower fractal dimension. Healthy bone exhibits a dense and well-organized fractal dimension, while osteoporotic bone shows an irregular dimension. Finally, the texture feature is extracted to identify the spatial information in an image.


$$\begin{array}{l} TX=\frac{\sum_{i}\sum_{j}\left(p_{i}-\mu_{i}\right)\left(p_{j}-\mu_{j}\right)}{\sigma_{i}\sigma_{j}} \end{array}$$ (16)

In the above Equation 16, where *TX* indicates the texture feature, μ_*i*_ and μ_*j*_ are the mean of the pixels *p*_*i*_, *p*_*j*_, σ_*i*_, and σ_*j*_ denotes a deviation of the pixels.

Transfer the extracted features to the third hidden layer for classifying input images as normal, Osteopenia, or osteoporosis. The Rand similarity index is used to evaluate the similarity between the extracted features and the test feature values for normal, Osteopenia, and osteoporosis.


$$\begin{array}{l} SI=1-\frac{\left|F_{\text{Ext}}\Delta F_{\text{tes}}\right|}{n} \end{array}$$ (17)

In the above Equation 17, where *SI* indicates a rand similarity index, *F*_*Ext*_ denotes extracted features, *F*_*tes*_ indicates testing features, *n* denotes the number of images, *F*_*Ext*_Δ*F*_*tes*_ indicates a variation between the two features. The similarity *RS* returns a value from 0 to 1. The high similarity value indicates that the given input image belongs to a particular class. Error rate *err* is estimated for each classification outcome in the following Equation 18.


$$\begin{array}{l} \text{err}={\left(Z_{\text{actual}}-Z_{\text{predicted}}\right)}^{2} \end{array}$$ (18)

Where *Z*_*actual*_ represents the true classification results and *Z*_*predicted*_ represents the predicted classification results obtained from the model. To optimize hyperparameters, the accuracy of osteoporosis classification was enhanced using an error back-propagation scheme during fine-tuning. The weight is updated with less error by the stochastic gradient function is given in the following Equation 19.


$$\begin{array}{l} w_{\text{upd}}=w_{t}-\eta \left[\frac{\partial \text{err}}{\partial w_{t}}\right] \end{array}$$ (19)

Where updated weight is *w*_*upd*_, present weight is *w*_*t*_, learning rate (η < 1) is η. A larger η allows *DL* to learn more quickly than a smaller value; the partial derivative of the error *err* with respect to *w*_*t*_ is $\left[\frac{\partial \text{err}}{\partial w_{t}}\right]$.

To minimize training and validation errors, the Gradient Divergence Crow Search Tuning algorithm is implemented. This nature-inspired, population-based metaheuristic method is inspired by the intelligent behavior of crows. These birds perform a collaborative search for food. In the context of the algorithm, each crow is related to a potential set of weights, while the food sources are related to the fitness function, which is evaluated based on the associated error values. Initialize the population of the crows (i.e., weights) *w*_*r*_ in the search space is given in the following Equation 20.


$$\begin{array}{l} w_{r}=\left\{w_{1},w_{2},w_{3},\ldots ,w_{r}\right\} \end{array}$$ (20)

After initialization, each crow's fitness (weight) is measured relative to the error rate.


$$\begin{array}{l} F\left(w\right)=\arg\min\text{err} \end{array}$$ (21)

In the above Equation 21, where *F*(*w*) indicates a fitness function, *argmin* represents the argument of the minimum function, and *err* refers to an error rate. Based on fitness value, the current best crow is selected. Then the position of the best individual is updated as follows,


$$\begin{array}{l} X_{t+1}=X_{t}+R\cdot L\cdot \left[0.5\cdot |X_{\text{best}}-X_{t}|\right] \end{array}$$ (22)

In the above Equation 22, where *X*_*t*+1_ denotes an updated position of the crow, *X*_*t*_ indicates a previous position of the crow, *R* indicates a random number ∈[0, 1], and *L* refers to the flight length for great influence on searching ability. A smaller value of *L* leads to local search, while a larger value of *L* leads to global search. *X*_*best*_ refers to the best position of the crow, 0.5|*X*_*best*_−*X*_*t*_| refers to a Jensen-Shannon divergence function. After that, the possibility of crowding is verified based on fitness.


$$\begin{array}{l} T=\{\begin{array}{ll} \text{Select }X_{t+1}\text{ as best solution}, & \text{if }F\left(X_{t+1}\right)>F\left(X_{t}\right)\\ \text{Select }X_{t}\text{ as best solution}, & \text{otherwise} \end{array} \end{array}$$ (23)

In the above Equation 23, where *T* indicates a verification result. If the fitness of the updated positions *F*(*X*_*t*+1_) is higher than the fitness of the previous position *F*(*X*_*t*_), the updated position is considered an optimal solution. Otherwise, the previous position *X*_*t*_ is preserved as the optimal solution. It repeated, awaiting the highest iterations to arrive. The optimal weight is identified and used to minimize error and increase classification accuracy. Therefore, accurate multi-class classification outcomes are obtained at the output layer using a softmax activation function.


$$\begin{array}{l} Y=\varphi_{\text{soft}}\left(h_{t}\cdot w_{ho}\right) \end{array}$$ (24)

In the above Equation 24, where *Y* indicates an output of classification, φ_soft_ indicates the softmax activation function, hidden layer output is *h*_*t*_, and the weight among the hidden as well as the output layer is *w*_*ho*_. The softmax activation function is employed for multiclass classification. The algorithmic process of the Gradient Divergence Tuned Deep Belief Network (DBN) model is given below.

Algorithm 2 presents a Gradient divergence-tuned deep belief network aimed at improving osteoporosis disease prediction accuracy while minimizing error rates. For each data input image, initial weights and biases are assigned to the input layer. The data then flows through hidden layers. Preprocessing techniques are applied to enhance image quality. Extract the most relevant features, edge, structure, and texture in hidden layer 2, thereby reducing the input dimensionality. Classification is performed in hidden layer 3, which evaluates the similarity between training and testing features using the Rand index to obtain the predictions. Following the classification stage, the Gradient Divergence Crow Search algorithm is employed for fine-tuning. This begins by initializing a set of weights and distributing a population of crow in the search space. Fitness is determined by classification error, and positions are updated iteratively across consecutive generations. This optimization process proceeds until a set number of iterations is completed. Finally, an optimal set of weights is determined that reduces the error. The multiclass prediction output is generated using the Softmax function, which further minimizes the classification error at the output layer.

Algorithm 2 Gradient divergence tuned deep belief network (Part 2: optimisation & output). 
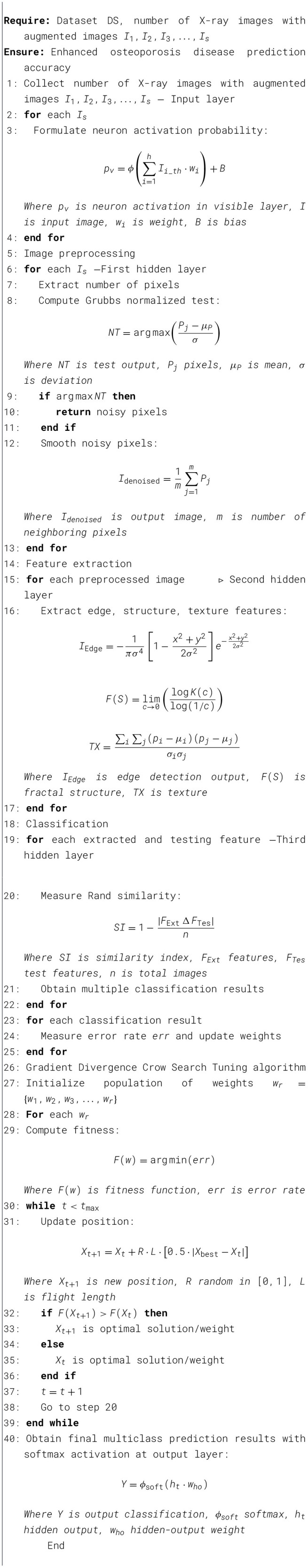

## 4 Experimental settings

The proposed TSFGDTDBN model, along with two existing methods [1], [2], [3], and [4], was implemented in Python with Jupyter Notebook using the Multi-Class Knee Osteoporosis X-Ray Dataset and the Knee X-ray Osteoporosis Database. The dataset used in this experiment is widely used in medical research, particularly for knee osteoporosis detection. It comprises medical imaging data, specifically X-ray images, along with associated patient information crucial for diagnosing knee osteoporosis. The dataset categorizes three classes. Detailed descriptions of images corresponding to each category are provided in Table 1.

**Figure 3:**
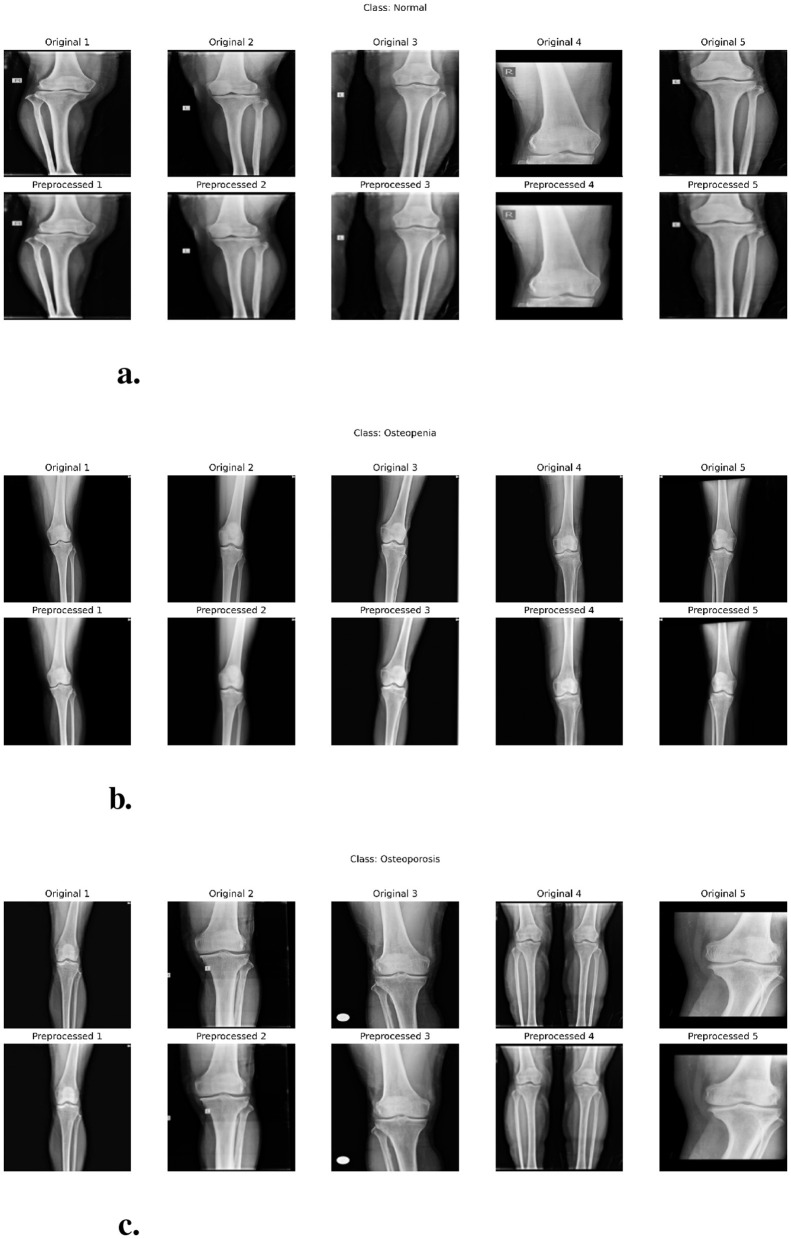
Preprocessing of X-ray images across different conditions. **(a)** Preprocessing using normal X-ray images using Multi-Class Knee Osteoporosis X-ray Dataset. **(b)** Preprocessing using Osteopenia X-ray images using Multi-Class Knee Osteoporosis X-ray Dataset. **(c)** Preprocessing using Osteoporosis X-ray images using Multi-Class Knee Osteoporosis X-ray Dataset.

Figures 3a–c illustrate the image preprocessing results using the Grubbs normalized linear box blur method. To enhance and preprocess the X-Ray images, the Grubbs normalized linear box blur method is used to effectively isolate and minimize noisy pixels. Grubbs' test is used to detect outlier pixel intensities. Once identified as statistical outliers representing noise or artifacts, these pixels are replaced or normalized. Standard box blur tends to smooth out high-frequency edges and fine bone textures. A normalized linear variation is used instead to retain important anatomical boundaries. In this way, image quality is boosted. As shown in Figure 3, the preprocessed images have higher image quality than the normal image.

**Figure 4:**
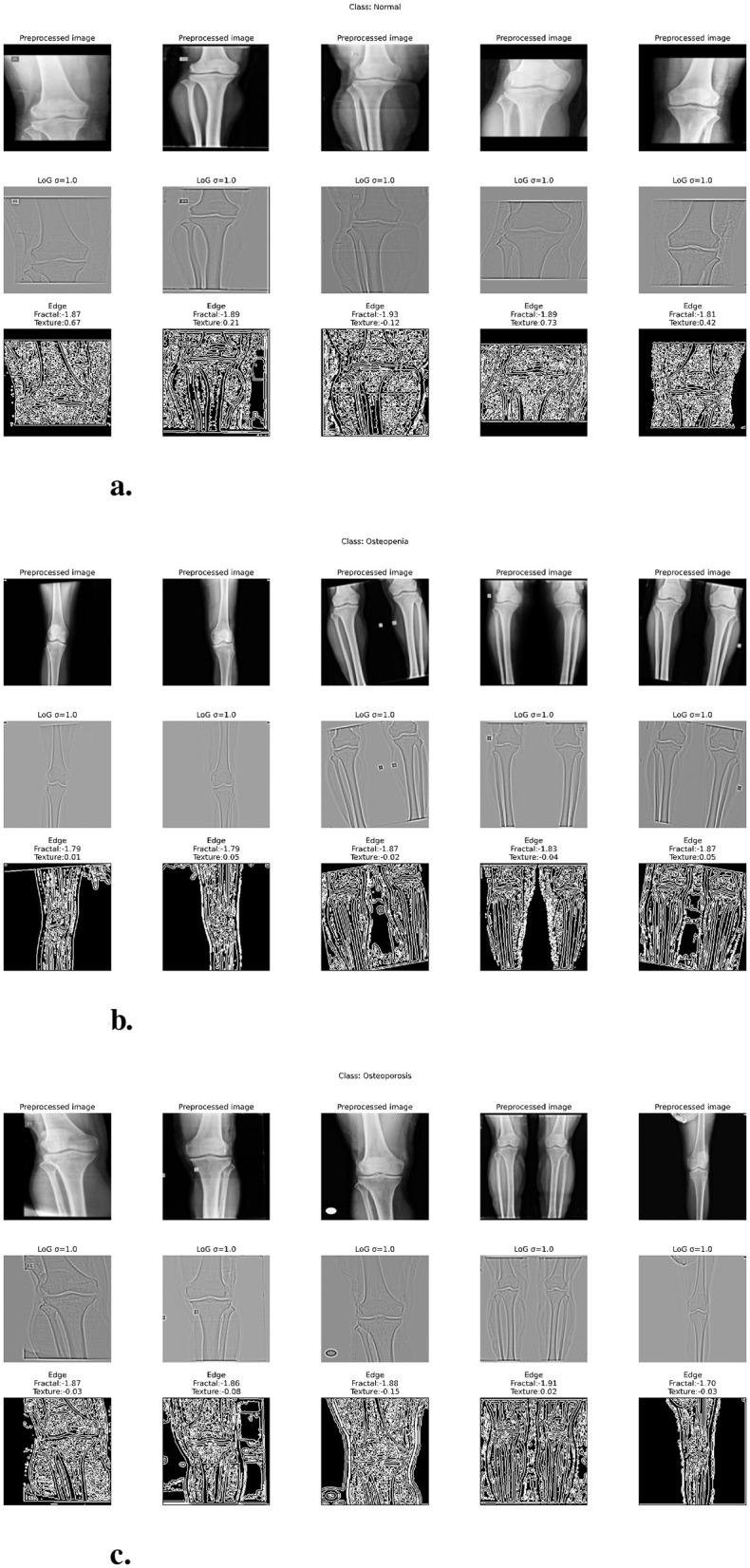
Illustrates the image feature extraction process applied to three diagnostic classes: Normal, Osteopenia, and Osteoporosis for the Multi-Class Knee Osteoporosis X-ray Dataset. **(a)** Edge, fractal structure, texture feature extraction using normal images. **(b)** Edge, fractal structure, texture feature extraction using Osteopenia images. **(c)** Edge, fractal structure, texture feature extraction using osteoporosis images.

Figures 4a–c illustrate image feature extraction for three different classes: normal, osteopenia, and osteoporosis. As shown in Figure 4, the Marr-Hildreth edge detector is applied for smoothing the edges of images. The Minkowski-Bouligand fractal geometric method is applied to evaluate the fractal dimension and characterize the complexity of bone structures in X-ray images for accurate osteoporosis detection. The texture feature is used to analyze the spatial relationships between pixels.

**Figure 5:**
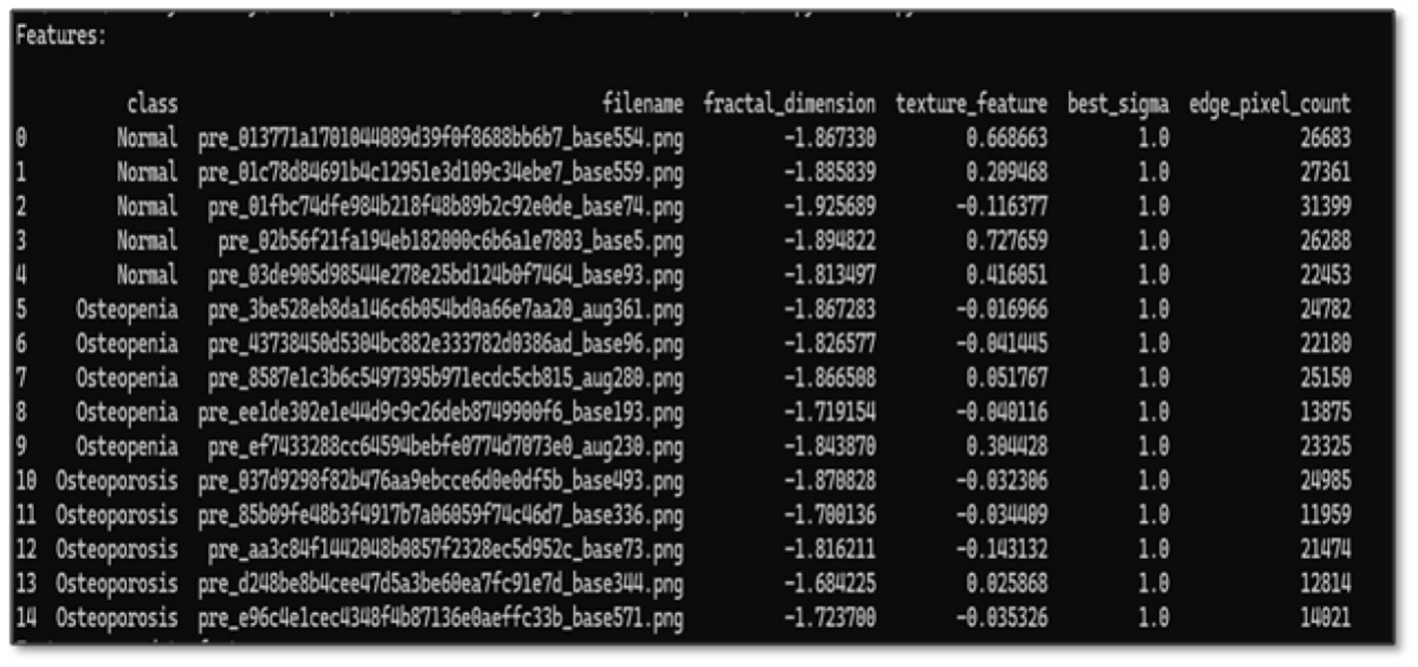
Combined feature extraction results for all images using Multi-Class Knee Osteoporosis X-ray dataset.

Figure 5 shows the combined image feature-extraction results for three classes: normal, osteopenia, and osteoporosis.

**Figure 6:**
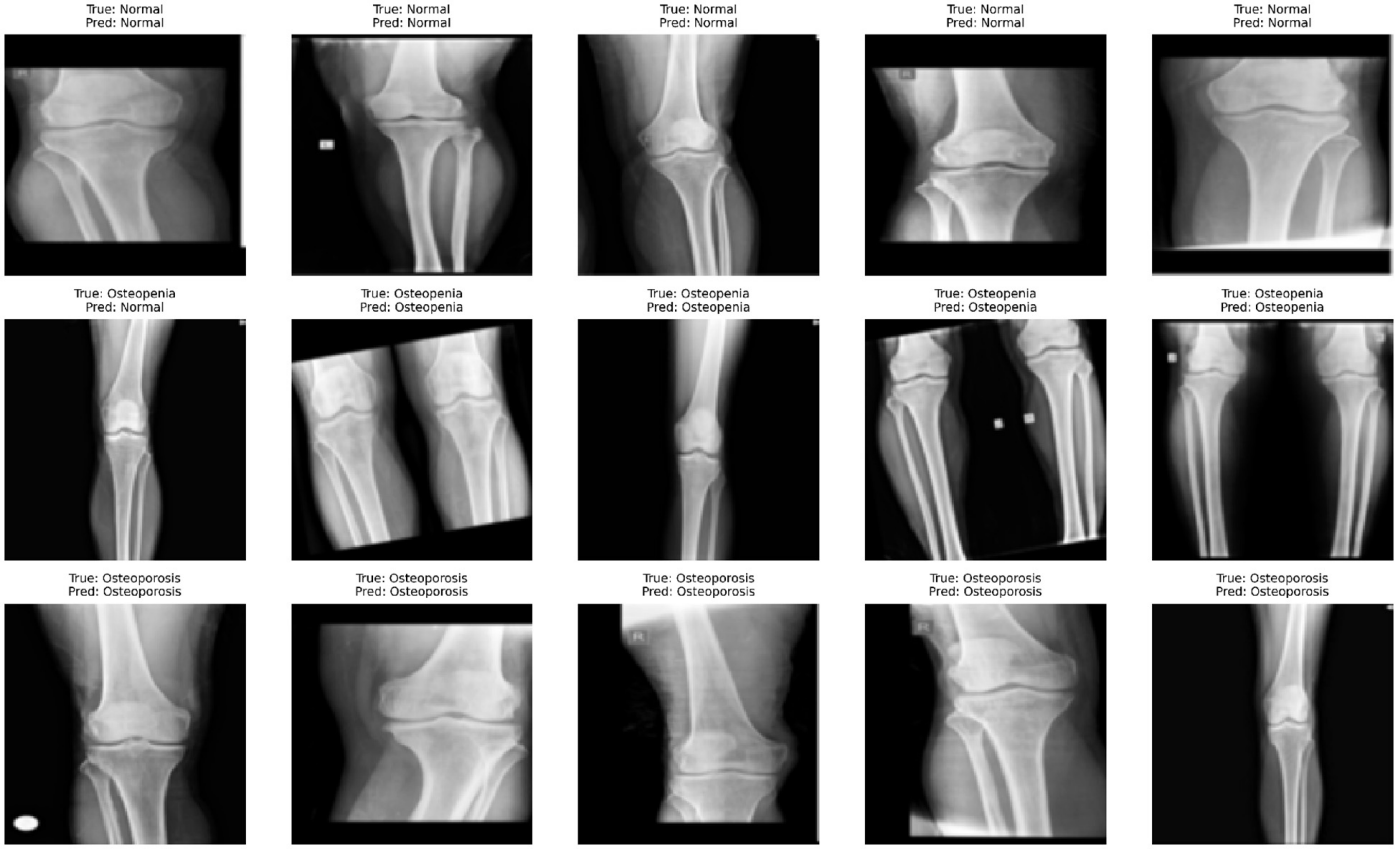
Classification results of the TSFGDTDBN model for Multi-Class Knee Osteoporosis X-ray Dataset.

**Figure 7:**
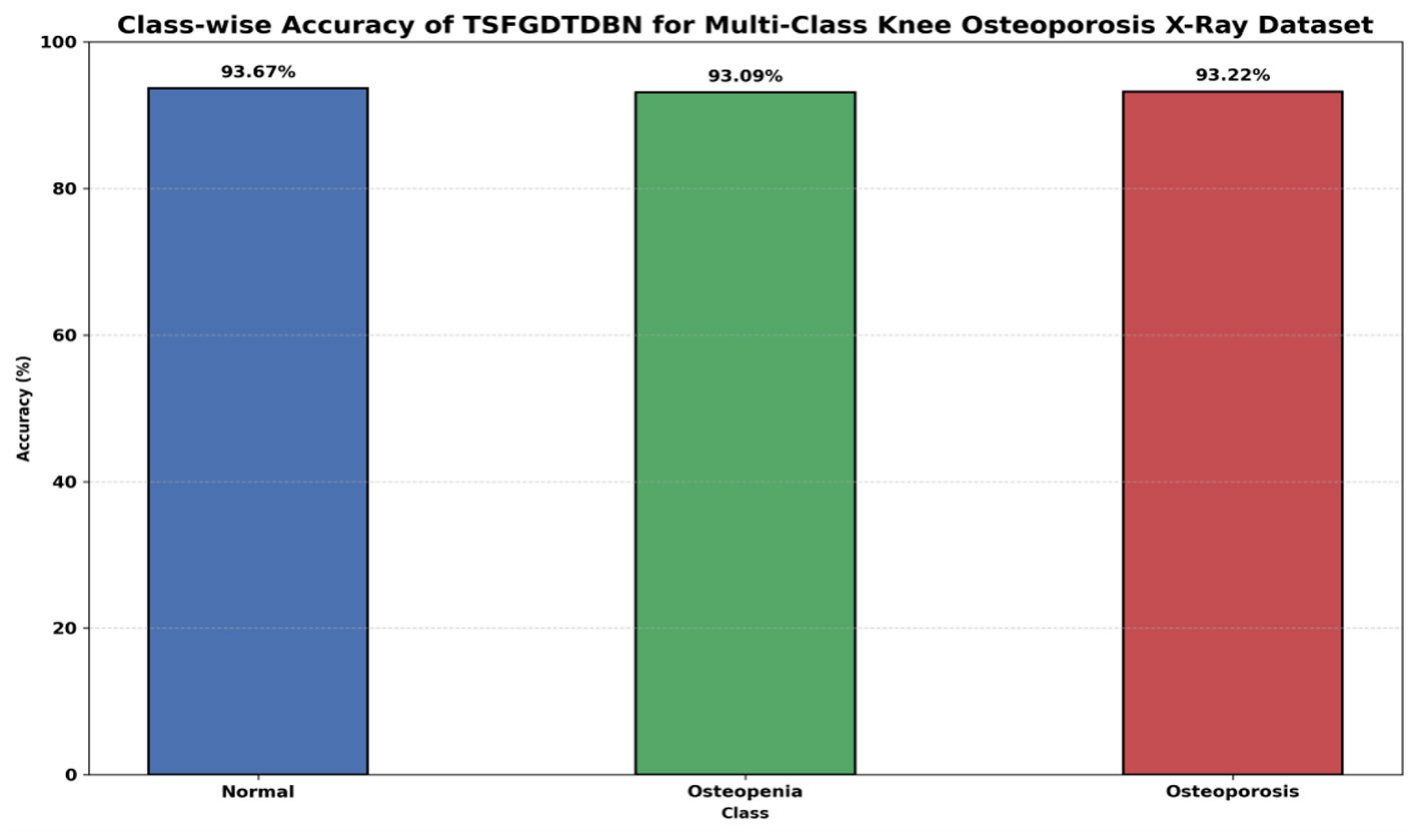
Class-wise accuracy performance for TSFGDTDBN using Multi-Class Knee Osteoporosis X-ray Dataset.

Figure 6 illustrates the classification of normal, osteopenia, and osteoporosis images using the Gradient Divergence DBN. Finally, disease classification is performed using the extracted features, with similarity measured via the Rand index. Based on the random similarity function, images of normal, Osteopenia, and Osteoporosis are classified. Figure 7 illustrates the class-wise accuracy performance of the proposed TSFGDTDBN model on the multi-class knee osteoporosis X-ray dataset. The model achieved 93.67% accuracy for Normal cases, 93.09% for Osteopenia, and 93.22% for Osteoporosis, demonstrating balanced classification capability across different severity levels.

## 5 Overall comparison analysis

This section presents TSFGDTDBN by comparison with existing methods, DenseNet201 with transfer learning [1], VGG-19 [2], ViT [3], and Deep CNN [4] in several key factors. Results of these performance indicators are systematically analyzed and illustrated using both tables and graphical representations.

### 5.1 Performance analysis of prediction accuracy

It evaluates a model that accurately detects individuals affected by osteoporosis, Normal, and Osteopenia. This effectiveness is commonly evaluated using a range of classification metrics to differentiate between positive and negative cases. The accuracy is mathematically formulated as follows:


$$\begin{array}{l} PA=\left(\frac{TRP+TNP}{TRP+TNP+FPR+FNR}\right)\times 100 \end{array}$$ (25)

In the above Equation 25, where *PA* indicates a prediction accuracy, true positive is *TRP*, false positive is *FPR*, true negative is *TNP*, and false negative is *FNR*.

**Figure 8:**
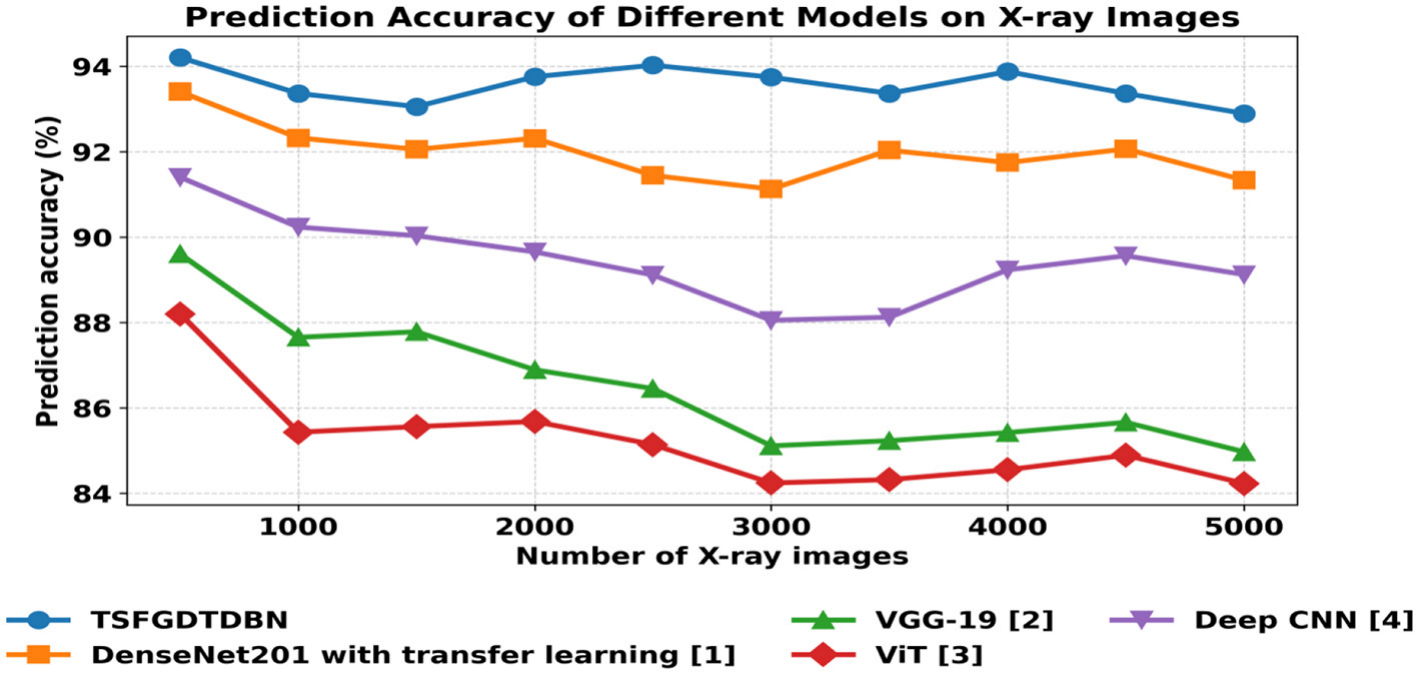
Performance analysis of prediction accuracy using Multi-Class Knee Osteoporosis X-ray Dataset.

Table 2 and Figure 8 illustrate a comparative analysis of prediction accuracy using five approaches, namely the proposed TSFGDTDBN model in comparison with existing methods, DenseNet201 with transfer learning [1], VGG-19 [2], ViT [3], and Deep CNN [4] for the Multi-Class Knee Osteoporosis X-Ray Dataset. X-ray images are denoted on the horizontal axis, while the vertical axis shows the corresponding prediction accuracy. Among the three methods, the TSFGDTDBN model consistently demonstrates superior predictive accuracy. For example, the TSFGDTDBN model achieved an accuracy of 94.2%, whereas the [1], [2], [3], and [4] approaches achieved 93.4%, 89.6%, 88.2%, and 91.4% accuracy, assuming 500 images. As the image count increases, varying results were observed across all methods, enabling a detailed performance comparison. From the results, the TSFGDTDBN model shows an accuracy improvement of approximately 2%, 8%, 10%, and 4% over [1], [2], [3], and [4]. This enhancement is achieved by integrating the Gradient Divergence Tuned DBN, which analyzes features, edges, shapes, and textures in input images using the rand similarity index function. Based on the random similarity function, normal, Osteopenia, and Osteoporosis are predicted with better accuracy.

**Table 2:** Comparison of prediction accuracy for Multi-Class Knee Osteoporosis X-ray Dataset.

Number of X-ray images	TSFGDTDBN	DenseNet201 with transfer learning [1]	VGG-19 [2]	ViT [3]	Deep CNN [4]
500	94.2	93.4	89.6	88.2	91.4
1,000	93.36	92.32	87.65	85.43	90.23
1,500	93.05	92.05	87.78	85.56	90.03
2,000	93.75	92.31	86.89	85.68	89.65
2,500	94.02	91.44	86.45	85.14	89.11
3,000	93.74	91.12	85.11	84.24	88.05
3,500	93.36	92.03	85.23	84.32	88.12
4,000	93.87	91.74	85.42	84.55	89.23
4,500	93.36	92.06	85.66	84.89	89.56
5,000	92.89	91.33	84.97	84.23	89.12

### 5.2 Performance analysis of precision

It refers to a measure of classification model efficiency in correctly predicting each specific class, particularly the positive cases. In a multi-class classification problem with Normal, Osteopenia, and Osteoporosis categories, this metric is calculated separately for each class. PC represents the percentage of the TRP of the sum of predictions is given in the following Equation 26


$$\begin{array}{c} PC=\left(\frac{TRP}{TRP+FLP}\right)\times 100 \end{array}$$ (26)

**Figure 9:**
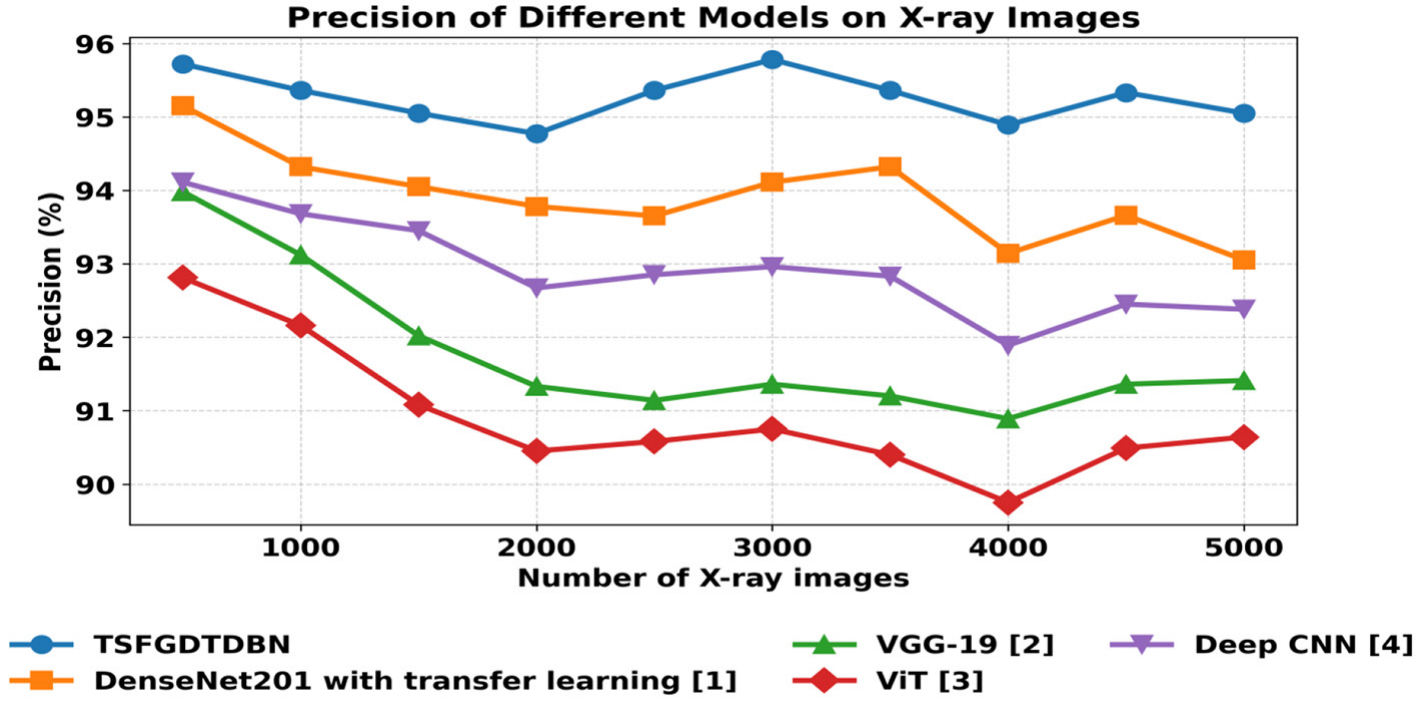
Performance analysis of precision using Multi-Class Knee Osteoporosis X-ray Dataset.

Table 3 and Figure 9 present a precision analysis across image counts from 500 to 5,000, using the Multi-Class Knee Osteoporosis X-Ray Dataset. The performance assessment involves five methods: the TSFGDTDBN model compared with existing methods: DenseNet201 with transfer learning [1], VGG-19 [2], ViT [3], and Deep CNN [4]. The experimental results indicate that the TSFGDTDBN model consistently outperforms the other two methods. For instance, let us consider a sample of 500 images: the TSFGDTDBN model achieved a precision of 95.72%, while [1], [2], [3], and [4] observed as 95.11%, 93.98%, 92.81%, and 94.11%, respectively. As the number of images increased, each method produced varying levels of precision. Comparative analysis shows that the TSFGDTDBN model improves precision by 2%, 4%, 5%, and 3% over [1], [2], [3], and [4]. This improvement is achieved by integrating the rand similarity index with an Optimized Tuning Deep Belief Network, enabling more efficient feature analysis. Moreover, the model incorporates the stochastic gradient algorithm for weight updates. The crow search tuning algorithm is designed to detect the optimal weight that significantly reduces classification errors and enhances performance by increasing the true positive rate while minimizing false positives.

**Table 3:** Comparison of precision for Multi-Class Knee Osteoporosis X-Ray Dataset.

Number of X-ray images	TSFGDTDBN	DenseNet201 with transfer learning [1]	VGG-19 [2]	ViT [3]	Deep CNN [4]
500	95.72	95.15	93.98	92.81	94.11
1,000	95.36	94.32	93.12	92.16	93.68
1,500	95.05	94.05	92.02	91.08	93.45
2,000	94.77	93.78	91.33	90.45	92.67
2,500	95.36	93.65	91.14	90.58	92.85
3,000	95.78	94.11	91.36	90.75	92.96
3,500	95.36	94.32	91.20	90.40	92.83
4,000	94.89	93.14	90.89	89.75	91.89
4,500	95.33	93.66	91.36	90.49	92.45
5,000	95.05	93.05	91.41	90.64	92.38

### 5.3 Performance analysis of recall

It is also referred to as sensitivity, and it evaluates a classification model's ability to correctly detect all actual positive instances. Equation for performance analysis for recall is given below in the Equation 27.


$$\begin{array}{c} RC=\left(\frac{TRP}{TRP+FLN}\right)\times 100 \end{array}$$ (27)

**Figure 10:**
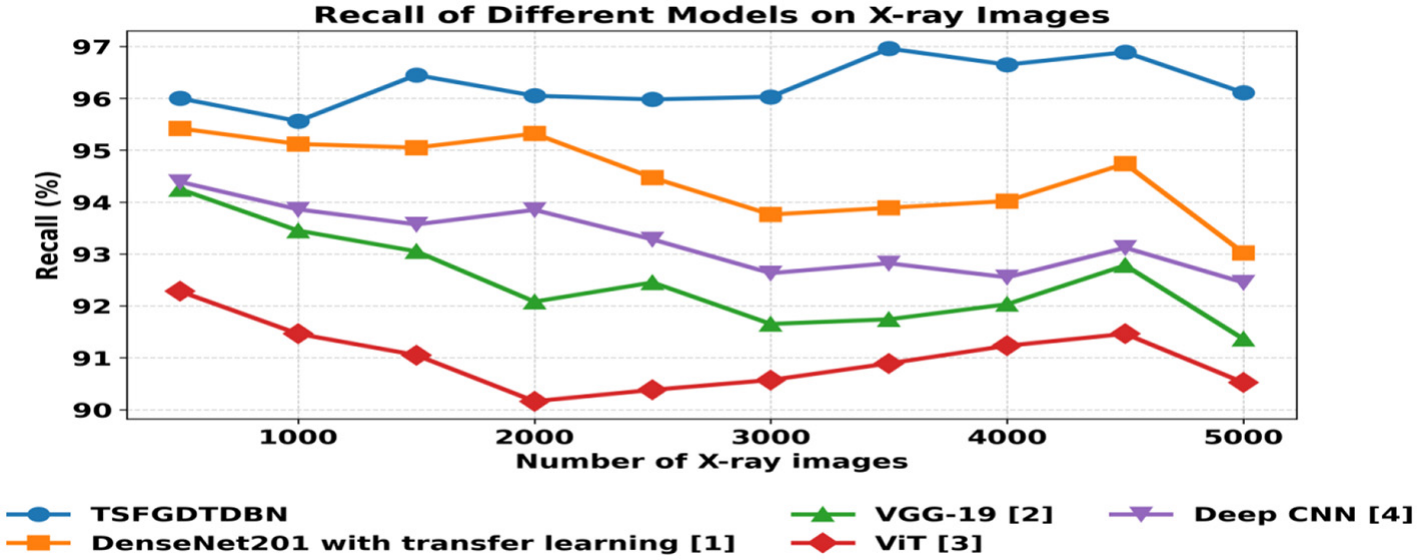
Performance analysis of recall.

Table 4 and Figure 10 present the analysis of recall performance across different image quantities, ranging from 500 to 5,000, using the Multi-Class Knee Osteoporosis X-Ray Dataset. The recall metric was evaluated using five methods: the proposed TSFGDTDBN model and existing methods, namely DenseNet201 with transfer learning [1], VGG-19 [2], ViT [3], and Deep CNN [4]. TSFGDTDBN achieves the highest recall among the three models. For example, with a dataset of 500 images, the TSFGDTDBN model achieved a recall of 96%, whereas [1], [2], [3], and [4] observed 95.42%, 94.25%, 92.28%, and 94.39%, respectively. As the image count increased, recall values varied across methods, allowing for a comprehensive performance comparison. Overall, the TSFGDTDBN model demonstrated recall improvements of 2%, 4%, 6%, and 3% over [1], [2], [3], and [4], respectively. This enhancement is mainly due to the integration of the Optimized Tuning Deep Belief Network, which assists in more efficient feature assessment. Furthermore, the model incorporates the Gradient stochastic crow search algorithm to optimize weight updates, which helps minimize errors and boosts performance by increasing the true positive rate while reducing false negatives, thereby enhancing recall.

**Table 4:** Comparison of recall for Multi-Class Knee Osteoporosis X-Ray Dataset.

Number of X-ray images	TSFGDTDBN	DenseNet201 with transfer learning [1]	VGG-19 [2]	ViT [3]	Deep CNN [4]
500	96.00	95.42	94.25	92.28	94.39
1,000	95.56	95.12	93.45	91.46	93.86
1,500	96.45	95.05	93.05	91.05	93.57
2,000	96.05	95.32	92.08	90.16	93.85
2,500	95.98	94.47	92.45	90.38	93.28
3,000	96.03	93.76	91.65	90.57	92.63
3,500	96.96	93.89	91.74	90.89	92.82
4,000	96.65	94.02	92.03	91.23	92.55
4,500	96.89	94.74	92.78	91.46	93.12
5,000	96.11	93.02	91.36	90.52	92.45

### 5.4 Performance analysis of F1-score

It integrates the performance metrics of precision “PC” and recall “RC.” The F1-score is calculated as given below in Equation 28:


$$\begin{array}{c} \text{F1-score}=2\times \left(\frac{PC\times RC}{PC+RC}\right) \end{array}$$ (28)

**Figure 11:**
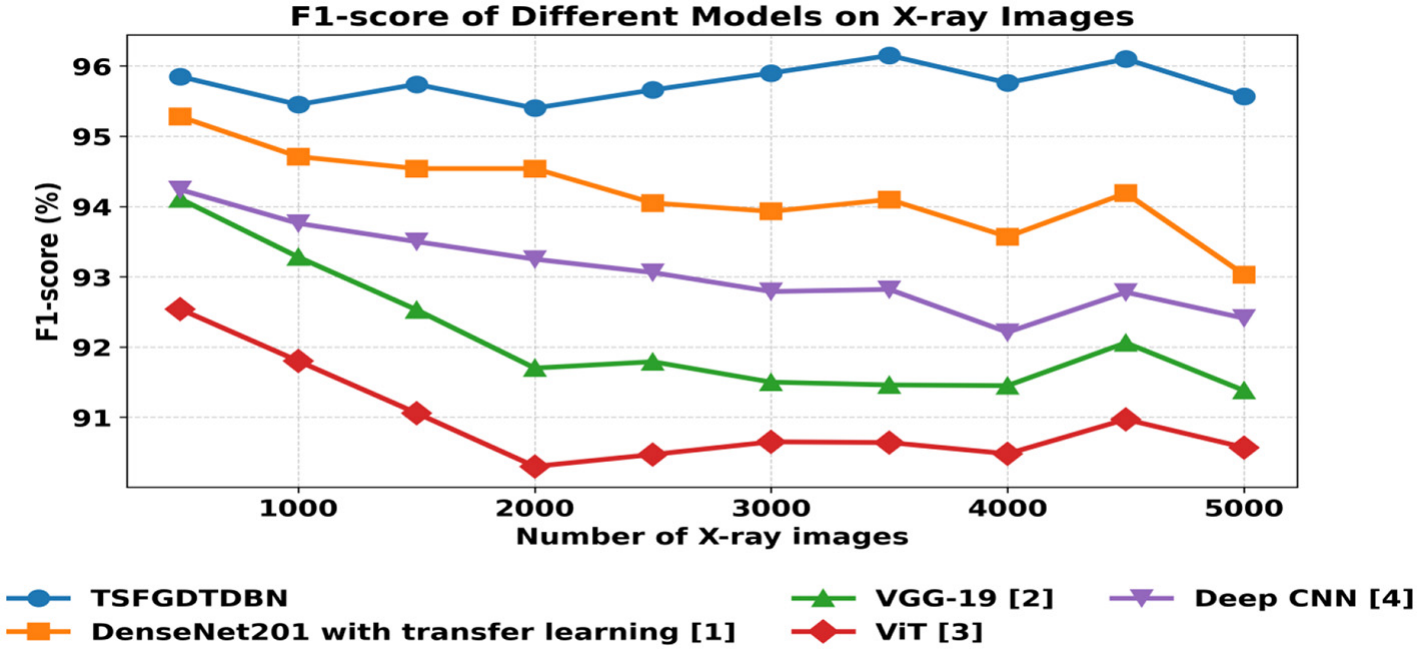
Performance analysis of F1-score using Multi-Class Knee Osteoporosis X-Ray Dataset.

Table 5 and Figure 11 illustrate the F1-score performance versus varying numbers of image samples, evaluated using five methods: the TSFGDTDBN model, DenseNet201 with transfer learning [1], VGG-19 [2], ViT [3], and Deep CNN [4] for the Multi-Class Knee Osteoporosis X-Ray Dataset. The F1-score was calculated using both precision and recall. The results clearly illustrate that the TSFGDTDBN model achieves a higher F1-score. This performance is achieved through the efficient use of a deep learning classifier strategy, which simultaneously improves both precision and recall in osteoporosis prediction tasks. As a result, the TSFGDTDBN model demonstrates a better ability to balance false positives and false negatives. Overall, the F1-score of the TSFGDTDBN model improved by 2%, 4%, 5%, and 3% over [1], [2], [3], and [4], respectively, highlighting its effectiveness in enhancing predictive accuracy.

**Table 5:** Comparison of F1-score for Multi-Class Knee Osteoporosis X-ray Dataset.

Number of X-ray images	TSFGDTDBN	DenseNet201 with transfer learning [1]	VGG-19 [2]	ViT [3]	Deep CNN [4]
500	95.85	95.28	94.11	92.54	94.24
1,000	95.45	94.71	93.28	91.80	93.76
1,500	95.74	94.54	92.53	91.06	93.50
2,000	95.40	94.54	91.70	90.30	93.25
2,500	95.66	94.05	91.79	90.47	93.06
3,000	95.90	93.93	91.50	90.65	92.79
3,500	96.15	94.10	91.46	90.64	92.82
4,000	95.76	93.57	91.45	90.48	92.21
4,500	96.10	94.19	92.06	90.97	92.78
5,000	95.57	93.03	91.38	90.57	92.41

### 5.5 Performance analysis of specificity

It measures to properly recognize negative images and is also called the true negative rate. The specificity is calculated as given in the following Equation 29.


$$\begin{array}{c} SPE=\left(\frac{TRN}{TRN+FLP}\right)\times 100 \end{array}$$ (29)

Where *SPE* denotes a specificity, *TRN* indicates a true negative, and *FLP* denotes a false positive rate.

**Figure 12:**
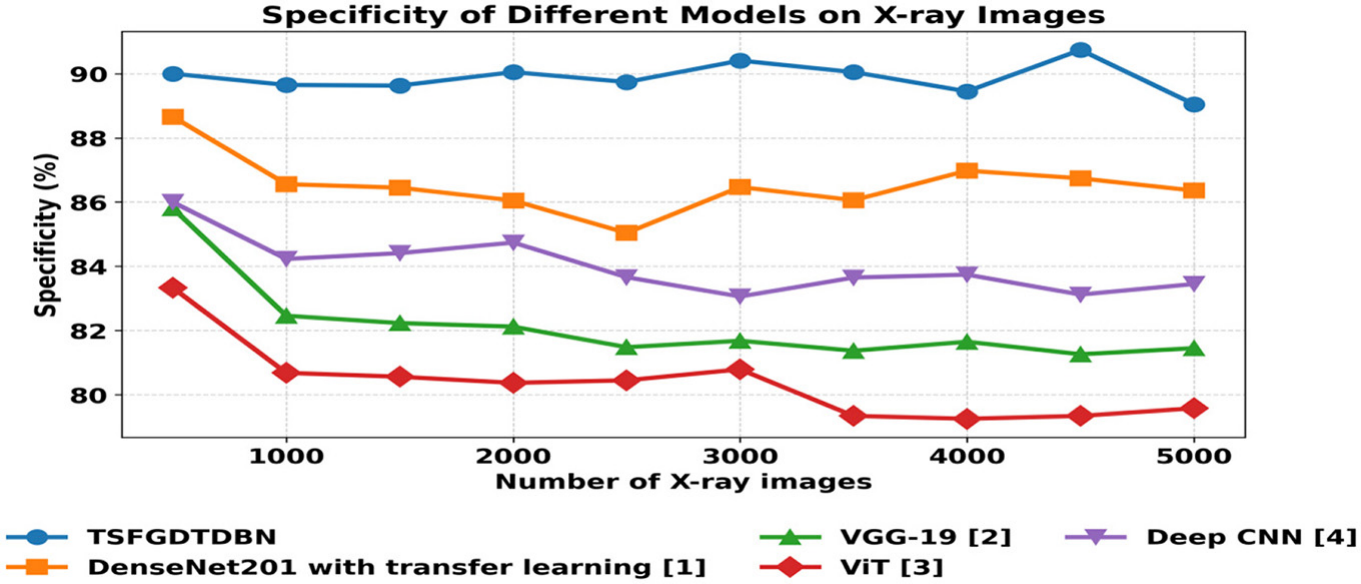
Performance analysis of specificity using Multi-Class Knee Osteoporosis X-ray Dataset.

Table 6 and Figure 12 present the specificity performance as a function of the number of image samples, ranging from 500 to 5,000, using the Multi-Class Knee Osteoporosis X-Ray Dataset. The experimental results indicate that the proposed TSFGDTDBN model consistently achieves higher specificity than the two existing deep learning methods. For example, 500 images are considered in the first iteration. The TSFGDTDBN model attained a specificity of 90%, outperforming the existing methods, which recorded values of 88.66%, 85.81%, 83.33%, and 86%, respectively. These results were analytically compared across different numbers of images to evaluate the F1 score for disease classification. The comparative analysis demonstrates that the TSFGDTDBN model improved specificity by 4%, 9%, 12%, and 7% compared with [1], [2], [3], and [4], respectively.

**Table 6:** Comparison of specificity for Multi-Class Knee Osteoporosis X-ray Dataset.

Number of X-ray images	TSFGDTDBN	DenseNet201 with transfer learning [1]	VGG-19 [2]	ViT [3]	Deep CNN [4]
500	90.00	88.66	85.81	83.33	86.00
1,000	89.65	86.56	82.46	80.68	84.23
1,500	89.63	86.45	82.23	80.56	84.41
2,000	90.05	86.05	82.12	80.37	84.74
2,500	89.74	85.04	81.48	80.45	83.66
3,000	90.41	86.47	81.68	80.79	83.06
3,500	90.05	86.06	81.37	79.34	83.65
4,000	89.45	86.98	81.65	79.25	83.74
4,500	90.74	86.74	81.26	79.34	83.12
5,000	89.05	86.36	81.45	79.58	83.45

### 5.6 Performance analysis of error rate

The error rate *ER* measures the proportion of incorrect predictions made by a classification model. A lower error rate signifies better model performance. It is calculated using the following Equation 30:


$$\begin{array}{c} ER=\left(\frac{FPR+FNR}{TRP+TNP+FPR+FNR}\right)\times 100 \end{array}$$ (30)

**Figure 13:**
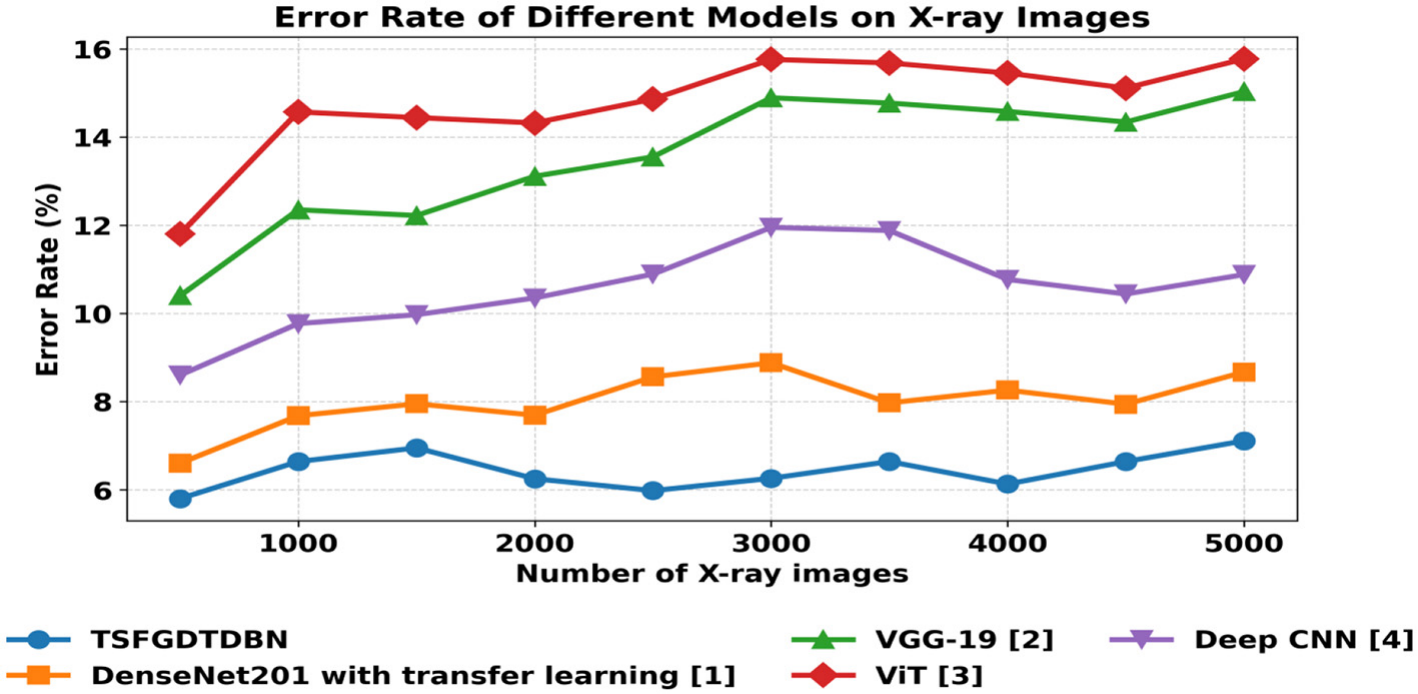
Performance analysis of error rate using Multi-Class Knee Osteoporosis X-ray Dataset.

Graphical outcome of error illustrated in Table 7 and Figure 13 using Multi-Class Knee Osteoporosis X-Ray Dataset. The results indicate that the TSFGDTDBN model considerably reduces the error rate compared to existing deep learning models. Finally, the comparison results indicate that the error rate using the TSFGDTDBN model is reduced by 19%, 52%, 56%, and 39% compared with [1], [2], [3], and [4], respectively. This reduction in error rate is achieved by applying the crow search optimization algorithm during the fine-tuning phase of the deep belief network model. This algorithm efficiently identifies optimal weights to enhance the model performance in osteoporosis disease prediction by reducing errors.

**Table 7:** Comparison of error rate for Multi-Class Knee Osteoporosis X-ray Dataset.

Number of X-ray images	TSFGDTDBN	DenseNet201 with transfer learning [1]	VGG-19 [2]	ViT [3]	Deep CNN [4]
500	5.80	6.60	10.40	11.80	8.60
1,000	6.64	7.68	12.35	14.57	9.77
1,500	6.95	7.95	12.22	14.44	9.97
2,000	6.25	7.69	13.11	14.32	10.35
2,500	5.98	8.56	13.55	14.86	10.89
3,000	6.26	8.88	14.89	15.76	11.95
3,500	6.64	7.97	14.77	15.68	11.88
4,000	6.13	8.26	14.58	15.45	10.77
4,500	6.64	7.94	14.34	15.11	10.44
5,000	7.11	8.67	15.03	15.77	10.88

### 5.7 Performance analysis of prediction time

It refers to the time needed to accurately predict sickness. Overall prediction time is measured as following Equation 31.


$$\begin{array}{c} PT=\overset{s}{\underset{i=1}{\sum }}I_{i}\cdot TM\left[P\right] \end{array}$$ (31)

Where *PT* indicates a prediction time, *s* is the image *I*_*i*_, and the Time to predict osteoporosis disease prediction of one image is *TM*[*P*]. *PT* is computed in milliseconds (*ms*).

**Figure 14:**
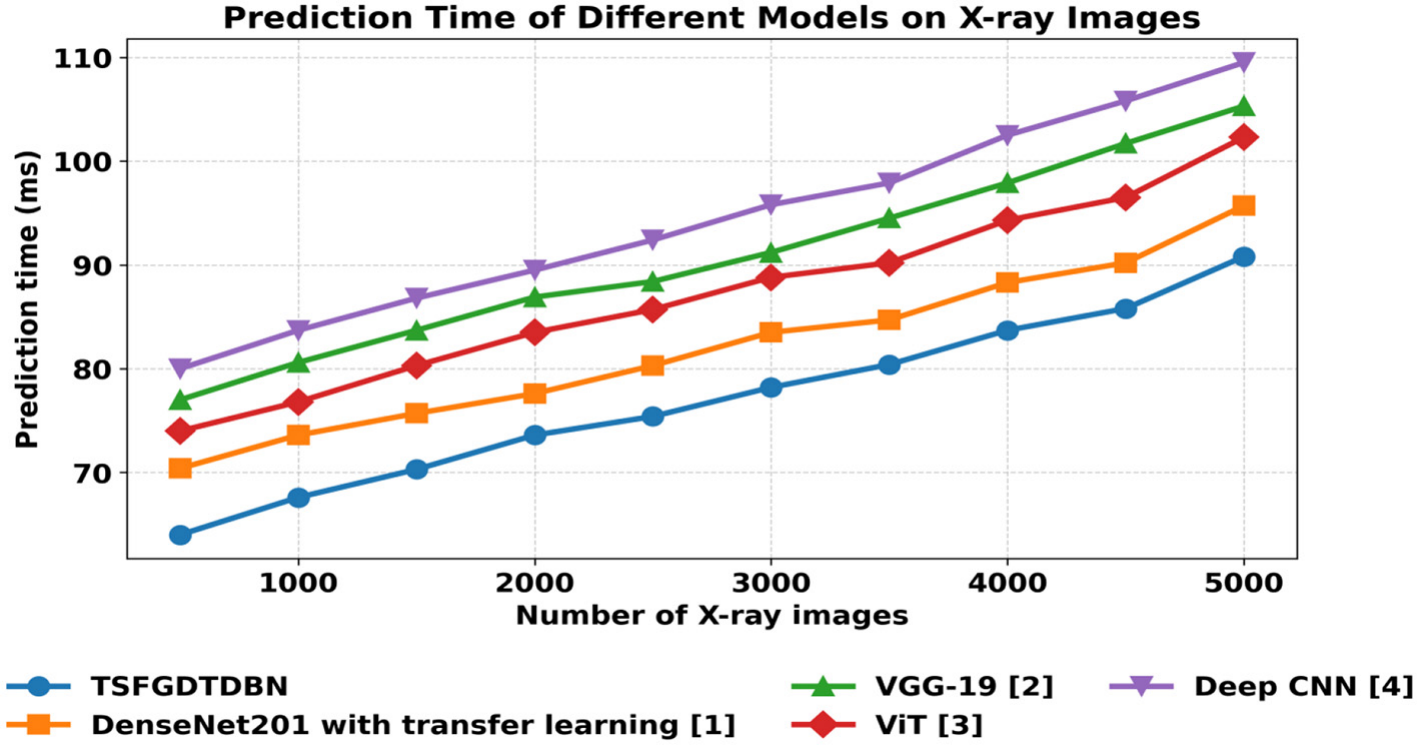
Performance analysis of prediction time using Multi-Class Knee Osteoporosis X-ray Dataset.

Table 8 and Figure 14 illustrate the prediction times for five methods, namely the TSFGDTDBN model, DenseNet201 with transfer learning [1], VGG-19 [2], ViT [3], and Deep CNN [4], on the Multi-Class Knee Osteoporosis X-Ray Dataset. Experiments for each method were conducted over 10 runs, using 5,000 images from the dataset. The figure shows that as the number of images increases, the time required to predict osteoporosis increases as well. However, the TSFGDTDBN model has a significantly shorter runtime than those of [1], [2], [3], and [4]. This is achieved by including effective image pre-processing and feature extraction. These steps contribute to a notable reduction in the time consumption for osteoporosis disease prediction. For instance, when tested on 500 images, the TSFGDTDBN model required only 64 ms for prediction, compared with 70.4 ms, 77 ms, 74 ms, and 80 ms for methods [1], [2], [3], and [4], respectively. Time consumption varied between different numbers of images for all methods. Finally, the overall findings highlight that the TSFGDTDBN model reduced prediction time by 6%, 15%, 12%, and 18% compared to the existing approaches.

**Table 8:** Comparison of prediction time for Multi-Class Knee Osteoporosis X-ray Dataset.

Number of X-ray images	TSFGDTDBN	DenseNet201 with transfer learning [1]	VGG-19 [2]	ViT [3]	Deep CNN [4]
500	64.0	70.4	77.0	74.0	80.0
1,000	67.6	73.6	80.6	76.8	83.7
1,500	70.3	75.7	83.7	80.3	86.8
2,000	73.6	77.6	86.9	83.5	89.5
2,500	75.4	80.3	88.4	85.7	92.4
3,000	78.2	83.5	91.2	88.8	95.8
3,500	80.4	84.7	94.5	90.2	97.9
4,000	83.7	88.3	97.9	94.3	102.5
4,500	85.8	90.2	101.7	96.5	105.8
5,000	90.8	95.7	105.3	102.3	109.5

### 5.8 Performance analysis of ROC and AUC

The Receiver Operating Characteristic (ROC) curve and the Area under the Curve (AUC) are generally derived from the Multi-Class Knee Osteoporosis X-Ray Dataset to assess the performance of chronic neuropathic pain detection. The ROC curve represents the correlation between the true positive rate (TPR) and the false positive rate (FPR) across dissimilar threshold values. Table 5 presents the experimental ROC-AUC results for the proposed TSFGDTDBN and existing methods [1], [2], [3], and [4].

**Figure 15:**
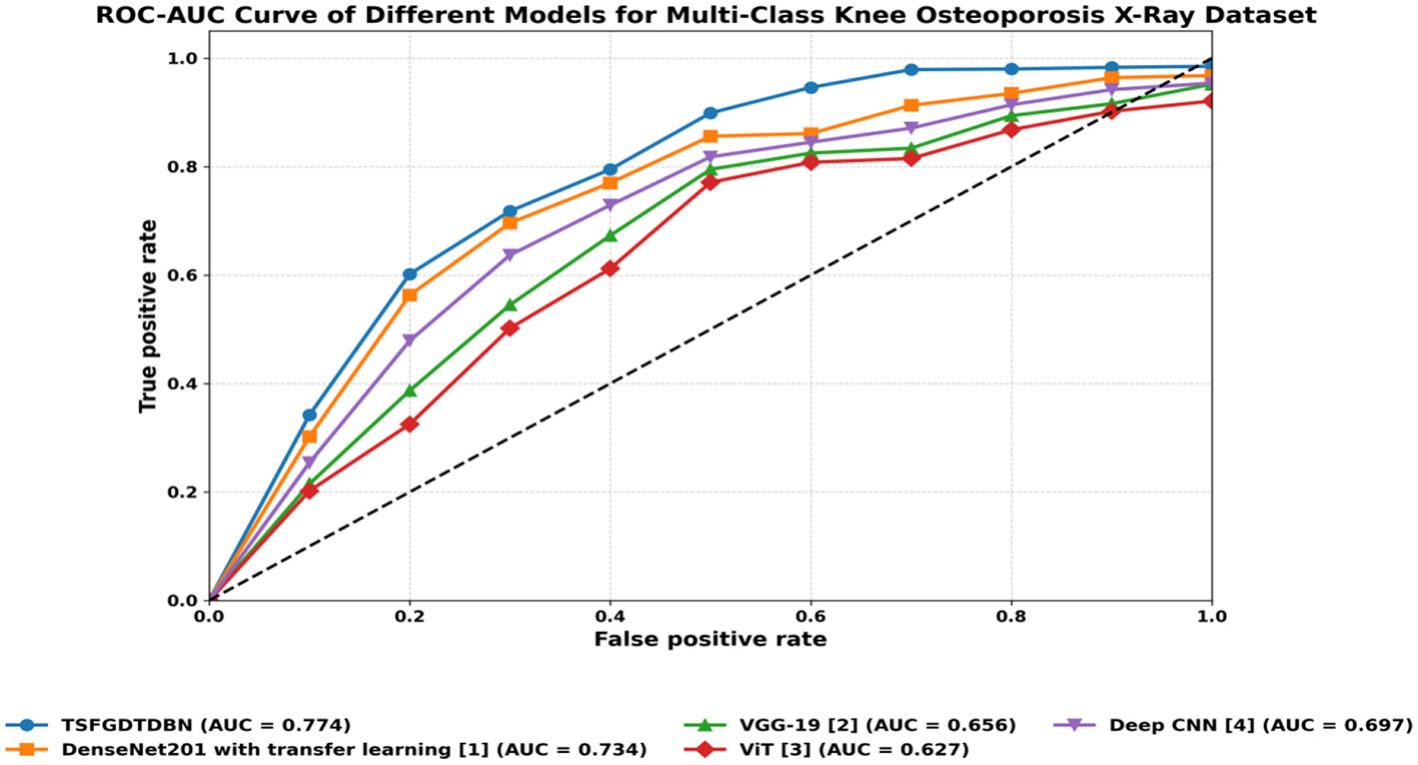
Performance analysis of ROC-AUC Curve results using Multi-Class Knee Osteoporosis X-ray Dataset.

In Table 9 and Figure 15, the Area under Curve (AUC) for different values ranging from 0 to 1. A value closer to 1 denotes a classifier that differentiates between normal and diseased patient data points. AUC value of 0.5 symbolizes a random opinion. A value below 0.5 indicates lower performance. The AUC values indicate that both the existing and proposed models yield better results. However, the proposed TSFGDTDBN method achieves a better outcome than existing methods [1], [2], [3], and [4].

**Table 9:** ROC-AUC results for Multi-Class Knee Osteoporosis X-Ray Dataset.

False positive Rate	True positive rate				
	TSFGDTDBN	DenseNet201 with transfer learning [1]	VGG-19 [2]	ViT [3]	Deep CNN [4]
0.0	0.000	0.000	0.000	0.000	0.000
0.1	0.342	0.302	0.215	0.202	0.254
0.2	0.602	0.563	0.387	0.325	0.479
0.3	0.718	0.696	0.545	0.502	0.637
0.4	0.795	0.777	0.673	0.612	0.729
0.5	0.899	0.856	0.795	0.771	0.818
0.6	0.946	0.861	0.825	0.808	0.845
0.7	0.979	0.913	0.834	0.815	0.874
0.8	0.980	0.935	0.894	0.868	0.924
0.9	0.983	0.916	0.902	0.912	0.941
1.0	0.985	0.968	0.952	0.921	0.954

### 5.9 Confusion matrix

The confusion matrix is a metric used on the Multi-Class Knee Osteoporosis X-ray Dataset to evaluate osteoporosis detection performance. The confusion matrix evaluates the presence or absence of disease against the actual results. In the experimental analysis, a dataset of 5,000 images is used. The confusion matrix is used to compare the performance of the proposed TSFGDTDBN.

**Figure 16:**
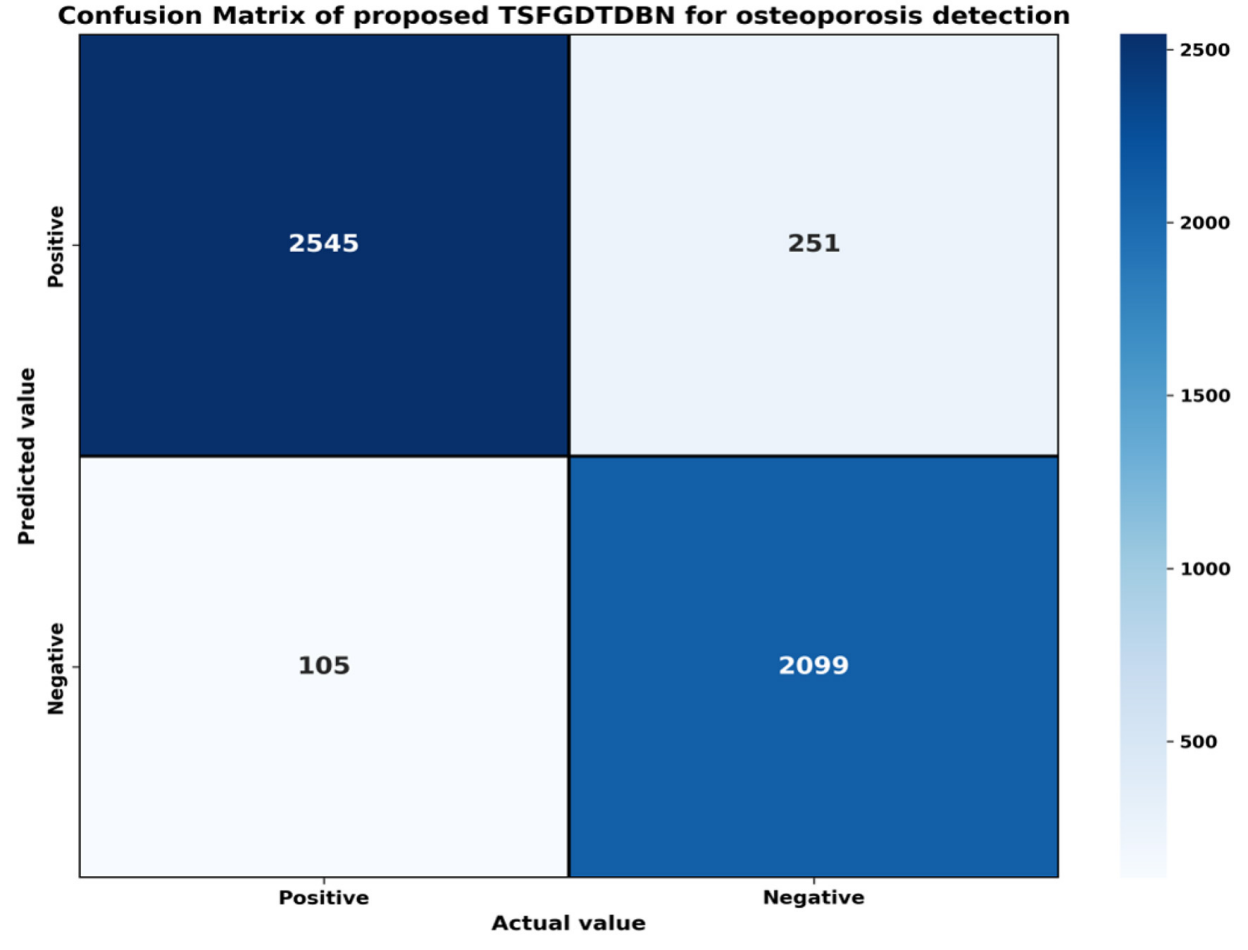
Experimentation results of confusion matrix of proposed TSFGDTDBN using Multi-Class Knee Osteoporosis X-ray Dataset.

Figure 16 illustrates the confusion matrix for the proposed TSFGDTDBN using the Multi-Class Knee Osteoporosis X-Ray Dataset. The matrix presents a diagrammatic representation of osteoporosis detection using 5,000 images. The confusion matrix summarizes the model's classification results using True Positives (TP), False Positives (FP), False Negatives (FN), and True Negatives (TN). Among the proposed and existing methods, the proposed TSFGDTDBN method achieved better performance, with higher true positive and true negative rates and lower false positive and false negative rates.

### 5.10 Ablation study for multi-class knee osteoporosis X-ray dataset

The ablation study is conducted on a Deep Belief Network to enhance overall performance. A Deep Belief Network is used to perform accurate osteoporosis prediction tasks. Deep Belief Network is combined with the Grubbs normalized linear box blur method, Marr–Hildreth edge detector, Minkowski Bouligand fractal geometric method, and Gradient Divergence Crow Search Tuning algorithm to enhance osteoporosis disease prediction performance. The assessment is carried out to improve the accuracy with minimum time consumption.

Table 10 shows the performance of the proposed method ablation study. DBN achieved an accuracy of 90.70%. The proposed TSFGDTDBN achieved 92.48% accuracy, 94.26% precision, 95.37% recall, 94.45% F1 score, and 88.75% specificity. The proposed TSFGDTDBN obtained a lower error rate of 6.1% and a prediction time of 75.43(ms) than other methods.

**Table 10:** Ablation study results on the Multi-Class Knee Osteoporosis X-ray Dataset.

Methods	Multi-Class Knee Osteoporosis X-ray Dataset						
	Accuracy (%)	Precision (%)	Recall (%)	F1 score (%)	Specificity (%)	Error rate (%)	Prediction time (ms)
DBN	90.70	91.85	94.22	93.46	83.68	8.3	89.78
DBN + Grubbs normalized linear box blur method	90.61	92.67	94.43	93.62	84.38	7.9	86.54
DBN + Marr–Hildreth edge detector	91.55	93.14	94.87	93.89	84.57	7.6	84.78
DBN + Minkowski Bouligand fractal geometric method	91.79	93.58	95.09	94.08	86.78	7.2	81.45
DBN + Gradient Divergensive Crow Search Tuning algorithm	92.14	94.13	95.14	94.12	88.12	6.8	78.78
DBN + Grubbs + Marr–Hildreth + Minkowski Bouligand + Gradient Divergensive Crow Search	92.48	94.26	95.37	94.45	88.75	6.1	75.43

### 5.11 Statistical test

The paired *t*-test is a statistical test used to predict osteoporosis. It is also called the dependent samples *t*-test. A paired *t*-test is conducted for TSFGDTDBN and the existing DenseNet201 with transfer learning [1], VGG-19 [2], ViT [3], and Deep CNN [4] on the Multi-Class Knee Osteoporosis X-Ray Dataset, based on accuracy.

**Figure 17:**
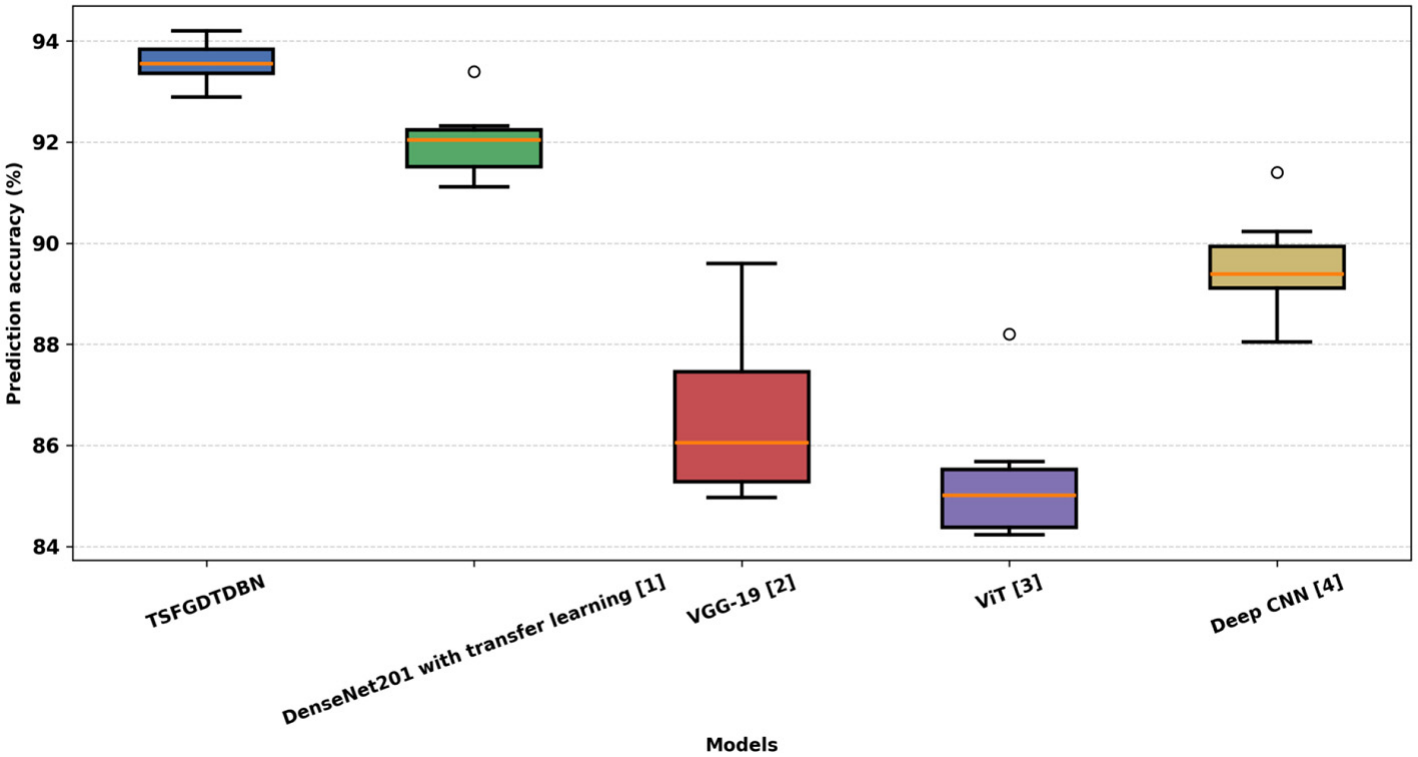
Paired *t*-tests outcomes.

In Table 11 and Figure 17, the paired t-test outcome is described. TSFGDTDBN achieved statistically significant improvements over DenseNet201 with transfer learning, VGG-19, ViT, and Deep CNN, with all *p*-values below 0.05. The highest t-statistic was observed against ViT, indicating the strongest performance difference. The paired *t*-test results show that TSFGDTDBN performed significantly better than all compared models.

**Table 11:** Statistical significance testing results for TSFGDTDBN compared with other models.

Comparison	t-statistic	*p*-value	Significance
TSFGDTDBN vs. DenseNet201 with transfer learning [1]	7.7082	0.00003	Significant
TSFGDTDBN vs. VGG-19 [2]	15.8124	0.00000	Significant
TSFGDTDBN vs. ViT [3]	25.4630	0.00000	Significant
TSFGDTDBN vs. Deep CNN [4]	13.1658	0.00000	Significant

### 5.12 Comparison of proposed and existing methods for Knee X-ray Osteoporosis Database

The analysis of the proposed TSFGDTDBN and the existing DenseNet201 with transfer learning [1], VGG-19 [2], ViT [3], and Deep CNN [4] is conducted in Python on the Knee X-ray Osteoporosis Database. Table 12 provides a detailed comparison between the proposed TSFGDTDBN and existing methods.

**Table 12:** Comparison of proposed and existing methods for Knee X-ray Osteoporosis Database.

Methods	Multi-Class Knee Osteoporosis X-ray Dataset						
	Accuracy (%)	Precision (%)	Recall (%)	F1 Score (%)	Specificity (%)	Error rate (%)	Prediction time (ms)
TSFGDTDBN	88.64	90.58	91.15	90.31	84.72	12.58	94.36
DenseNet201 with transfer learning	83.32	88.25	89.36	88.82	81.45	15.81	108.21
VGG-19	85.58	89.59	91.47	90.36	82.78	14.53	97.89
ViT	89.45	91.86	92.48	91.57	85.82	11.37	92.28
Deep CNN	91.36	92.37	94.29	93.16	87.43	8.90	81.35

Table 12 shows the performance of the proposed and existing methods for the Knee X-ray Osteoporosis Database. The proposed TSFGDTDBN achieved 91.36% accuracy, 92.37% precision, 94.29% recall, 93.16% F1 score, and 87.43% specificity. The proposed TSFGDTDBN obtained a lower error rate of 8.9% and a prediction time of 81.35(ms) than other methods.

### 5.13 Clinical relevance discussion

DBNs are probabilistic generative models used to extract complex, hidden features from high-dimensional medical data. They are used in clinical decision systems to analyze medical imaging (for feature extraction and segmentation) and to predict osteoporosis. Based on the results, the proposed TSFGDTDBN model achieves strong osteoporosis prediction performance, with high accuracy, precision, and recall, and a fast disease prediction time. The classification results are used for clinical decision support systems (CDSS) in real-time applications. The proposed TSFGDTDBN helps healthcare professionals diagnose osteoporosis and optimize bone health assessments with minimal resources. TSFGDTDBN is used to enable timely medical intervention and enhance preventive care.

## 6 Conclusion

Early detection and continuous monitoring of osteoporosis play a vital role in enhancing patient outcomes. The TSFGDTDBN model was developed to improve the accuracy of early osteoporosis prediction and reduce prediction time. The TSFGDTDBN model performs image preprocessing and feature extraction, thereby reducing the time required for osteoporosis prediction. The integration of preprocessing steps and sophisticated feature extraction enables the TSFGDTDBN model to achieve faster, more accurate osteoporosis prediction in medical diagnostics. The proposed deep belief network analyzes the extracted features and obtains multiple classification results at the output layer with minimal error. Several parameters are used to conduct the experimental assessment. TSFGDTDBN outperforms existing deep learning methods, delivering higher accuracy, lower error rates, and faster prediction times.

## Data Availability

The datasets were generated from “https://www.kaggle.com/datasets/mohamedgobara/multi-class-knee-osteoporosis-x-ray-dataset”, associated with a paper that is available and can also be accessed.
